# Site-Directed Fluorescence Approaches for Dynamic Structural Biology of Membrane Peptides and Proteins

**DOI:** 10.3389/fmolb.2019.00096

**Published:** 2019-09-25

**Authors:** H. Raghuraman, Satyaki Chatterjee, Anindita Das

**Affiliations:** Crystallography and Molecular Biology Division, Saha Institute of Nuclear Physics, Homi Bhabha National Institute, Kolkata, India

**Keywords:** membrane proteins, site-directed fluorescence, tryptophan, NBD and bimane fluorescence, REES, smFRET, membrane penetration depth, TrIQ

## Abstract

Membrane proteins mediate a number of cellular functions and are associated with several diseases and also play a crucial role in pathogenicity. Due to their importance in cellular structure and function, they are important drug targets for ~60% of drugs available in the market. Despite the technological advancement and recent successful outcomes in determining the high-resolution structural snapshot of membrane proteins, the mechanistic details underlining the complex functionalities of membrane proteins is least understood. This is largely due to lack of structural dynamics information pertaining to different functional states of membrane proteins in a membrane environment. Fluorescence spectroscopy is a widely used technique in the analysis of functionally-relevant structure and dynamics of membrane protein. This review is focused on various site-directed fluorescence (SDFL) approaches and their applications to explore structural information, conformational changes, hydration dynamics, and lipid-protein interactions of important classes of membrane proteins that include the pore-forming peptides/proteins, ion channels/transporters and G-protein coupled receptors.

## Introduction

Biological membranes are composed of heterogeneous mixtures of lipids and proteins which facilitate cellular compartmentalization for specialized functions. The biological membrane serves as a platform for many important functions like signal transduction, muscle contraction, ion transport, cell-contact, and recognition (Shai, 2001). Interestingly, one third of proteins produced by genomes of prokaryotes and eukaryotes are membrane proteins (Wallin and Heijne, 1998; Fagerberg et al., 2010). Further, membrane proteins are important drug targets for two-thirds of approved drugs—G-protein coupled receptors (GPCR) and ion channels represent the largest groups (Terstappen and Reggiani, 2001; Yildirim et al., 2007; Bakheet and Doig, 2009; Bull and Doig, 2015). Therefore, understanding the mechanistic details of the function of membrane proteins is crucial for biomedical research and drug discovery.

**Figure 1:**
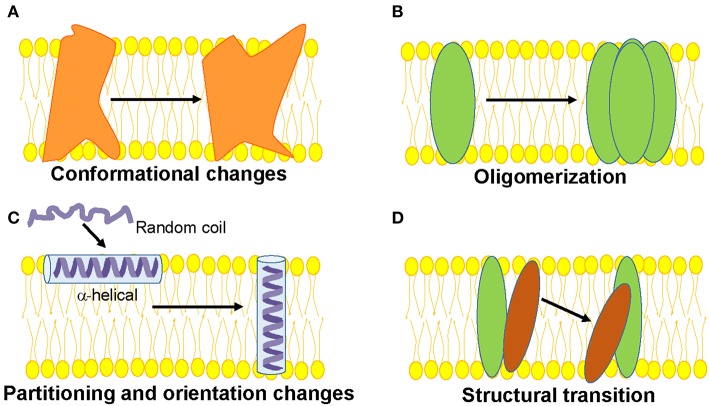
Schematic representations of functionally-relevant structural changes in membrane proteins are shown. **(A)** Conformational changes in response to a physiological signal such as voltage, ligand, pH, membrane tension etc. **(B)** Oligomerization involving protein-protein interactions, **(C)** Partitioning, folding and insertion of membrane proteins such as pore-forming proteins **(D)** Lipid-protein interactions resulting in structural transition.

Generally, the structure and function of membrane proteins is influenced by its membrane environment and the interactions within the structure (protein-protein interactions) and between lipids and proteins (lipid-protein interactions). Therefore, knowledge of how membranes shape protein structure with respect to its partitioning, change in orientation of α-helices, oligomerization, structural transition etc. is of vital importance to decipher the mechanism(s) of membrane protein function (Figure 1). Further, there exists significant differences between soluble and membrane proteins (Eilers et al., 2002; Zhou and Cross, 2013). For instance, the intrahelical hydrogen bonds are shorter in membrane proteins (Kim and Cross, 2002) and the backbone atoms of transmembrane helices experience less competition from water and hydrophilic side chains. In addition, the transmembrane helices of membrane proteins have significantly reduced highly polar (Asn and Gln) and charged (Arg and Lys) residues by a factor of ~3 (Eilers et al., 2002). A notable exception is the S4 helix of the voltage-sensing domains of voltage-gated ion channels and voltage-sensitive phosphatases (Jiang et al., 2003; Murata et al., 2005), in which there are several charged residues—mainly Arg and are called gating charges—present that help to generate electrical signaling in biology (Jiang et al., 2003; Catterall, 2010). Even the amino acids Gly and Pro, which are known as helix breakers, are present in high content throughout the transmembrane helices compared to helices from soluble proteins. Particularly, Gly plays an important role in facilitating interhelical interactions and folding stability of α-helical proteins.

**Figure 2:**
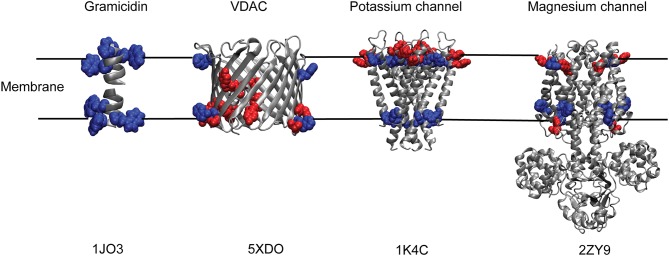
“Aromatic ring” formed by Trp and Tyr residues of membrane proteins at the membrane interface. Cartoon representations of Gramicidin A; human voltage-dependent anion channel, VDAC; potassium channel, KcsA and magnesium channel, MgtE with their respective tryptophan (blue) and tyrosine (red) residues depicting the membrane interfacial localization of the aromatic ring. Only the transmembrane Tyr and Trp residues are shown for MgtE. PDB IDs of the respective protein are indicated below each representation. See text for details.

Interestingly, transmembrane proteins have significantly higher content of Trp amino acids compared to water-soluble proteins (Schiffer et al., 1992; Eilers et al., 2002). Generally, Trp residues comprise ~1 mol% in soluble proteins, whereas the relative abundance of Trp is much higher (~3–7%) in most membrane proteins (Wallace and Janes, 1999). For instance, membrane proteins like bacteriorhodopsin, cytochrome oxidase, α-hemolysin, and KcsA potassium channel have ~3% of Trp residues; photosynthetic reaction center and maltoporin have ~4.5%; and light harvesting complex has as high as 7% Trp residues. Of course, there are exceptions to this high abundance of Trp residues in membrane proteins—MgtE magnesium channel has 1.3% of Trp residues that is comparable to soluble proteins, and OmpF, a beta-barrel porin, has only 0.6% of Trp residues. Another important difference is the distribution of Trp residues in soluble and membrane proteins. While Trp residues are distributed throughout the soluble protein structures (Chothia, 1976), it is well known that Trp (and Tyr) amino acids in many transmembrane proteins and peptides are not uniformly distributed and that they prefer to localize at the membrane interface, which accounts for almost half of the bilayer's thermal thickness (White and Wimley, 1994; Raghuraman et al., 2005). For example, crystal structures of membrane proteins like potassium channels, KcsA (Zhou et al., 2001) and KirBac1.1 (Kuo et al., 2003), MgtE magnesium channel (Hattori et al., 2009; Tomita et al., 2017), maltoporin (Schirmer et al., 1995), glycerol-conducting channel (Fu et al., 2000), and others have shown that Trp and Tyr residues seem to have an anchoring role by forming an “aromatic ring” around them at the membrane interface (Figure 2) and defines the hydrophobic length of transmembrane helices (Yau et al., 1998; Killian and von Heijne, 2000; Demmers et al., 2001; de Jesus and Allen, 2013). It is also worth noting that the distribution of aromatic residues in the membrane bilayer for β-barrel membrane proteins is different from those of α-helical membrane proteins. Although “aromatic ring” is formed in different families of both α-helical and β-barrel membrane proteins (see Figure 2), the “aromatic rings” from both sides of the membrane are closer together in β-barrel proteins (spacing of ~20 Å) compared to ~30 Å spacing in α-helical membrane proteins (Ulmschneider and Sansom, 2001). Contrary to the preferential localization of Trp residues in membrane proteins, the Trp is also localized at the hydrophobic core of the membrane in a few cases, e.g., KvAP voltage-sensing domain (Krepkiy et al., 2009) and transmembrane domain of human inositol requiring enzyme, IRE1α (Cho et al., 2019).

Despite technological advances in synchrotron radiation and recent excellent successes in structural determination of membrane proteins (Moraes et al., 2014; Hendrickson, 2016), the high-resolution molecular characterization of membrane proteins and peptides is still a great challenge due to various reasons pertaining to low extraction and yield of stable and functional proteins, complexity of source membranes (Carpenter et al., 2008) and difficulty in obtaining diffraction-grade crystals (Moraes et al., 2014). Although the first X-ray structure determination of a membrane protein was accomplished more than three decades back (Deisenhofer et al., 1985), the number of unique membrane protein structures solved is only ~2% of all solved protein structures (Feroz et al., 2018). Interestingly, with the exception of bacteriorhodopsin (Grigorieff et al., 1996), structural characterization of all membrane proteins have been done in non-native, membrane-mimetic systems. Through X-ray diffraction data, the detailed static structural information at high resolution can be obtained, however, the dynamic information is usually lacking. However, it is well recognized that structural dynamics play important roles in the function of proteins. Furthermore, high-resolution Nuclear Magnetic Resonance (NMR) methods, which are sophisticated to obtain the structure and dynamics of proteins, have limited applications for membrane proteins due to slow reorientation times of molecules in membranes (Opella, 1997).

Classic techniques used to determine the structure of protein like X-ray crystallography, NMR, and cryo-EM subject the protein to non-native or non-physiological conditions (specifically X-ray crystallography). Further, the above techniques require large amounts of pure and stable protein, which are often difficult to obtain in case of membrane proteins. Even after obtaining large amounts of pure, stable and functional protein, there is no guarantee of crystal formation that is needed for X-ray crystallography (Kaback and Wu, 1999). For these reasons, studies involving structural analyses of membrane proteins have employed various biophysical techniques mainly on spectroscopic approaches. Of these, fluorescence spectroscopic approaches are the most versatile and are used to asses both the structure and dynamics of membrane-bound proteins in their physiological conditions (Chattopadhyay and Raghuraman, 2004; Johnson, 2005).

## Why Site-Directed Fluorescence?

### Intrinsic Protein Fluorescence

Among the aromatic acids (Trp, Tyr, and Phe), intrinsic Trp fluorescence is the most widely used tool to monitor the changes in local structure and dynamics in proteins (Vivian and Callis, 2001; Raghuraman et al., 2005; Callis and Tusell, 2014). In case of membrane-active peptides and proteins such as the hemolytic peptide melittin (Raghuraman and Chattopadhyay, 2005, 2007a), cytochrome b5 (Ladokhin et al., 1991), γM4 transmembrane domain peptide of the nicotinic acetylcholine receptor (Barrantes et al., 2000; de Almeida et al., 2004), colicin E1 channel peptide (Tory and Merrill, 2002), pore-forming channel protein OmpF (Pattnaik et al., 1997), and the isolated voltage sensing domain of KvAP potassium channel (Krepkiy et al., 2009), intrinsic fluorescence from the single Trp residue has been used to monitor membrane partitioning, folding, orientation and topology. In addition, environment sensitivity of Trp fluorescence has been extensively used to characterize lipid-protein interactions in micelles and membranes (Raghuraman and Chattopadhyay, 2004a,b,c; Rawat et al., 2004; Raghuraman et al., 2006; Haldar et al., 2010b).

However, as mentioned earlier, membrane proteins contain higher content of Trp and hence the fluorescence data interpretation from multi-Trp proteins is generally complicated due to complex fluorescence processes and environment-sensitive nature of individual Trp residues (Eftink, 1991; Engelborghs, 2003). Further, light scattering from lipid vesicles is an important concern and care must be taken while analysing the Trp fluorescence of membrane peptides and proteins in membranes (Ladokhin et al., 2000). Although analyses of ensemble tryptophan fluorescence from multi-tryptophan containing membrane proteins is complicated, it provides information regarding overall structural integrity and functional organization of the protein (Chatterjee et al., 2019). However, the information related to site-specific dynamic changes that are relevant for the function of protein cannot be obtained. This concern can easily be avoided by using extrinsic fluorophores with excellent spectral properties. In this regard, an approach which combines the traditional site-directed mutagenesis (SDM) technique with attachment of extrinsic fluorophore to the single-site of interest in a site-specific manner is known as site-directed fluorescence labeling (Heuck and Johnson, 2002; Chattopadhyay and Raghuraman, 2004; Johnson, 2005).

### Site-Directed Fluorescence Labeling

The most widely used and faithful fluorophores to monitor conformational changes and protein backbone positions are cysteine-reactive labels (Heuck and Johnson, 2002; Johnson, 2005; Mansoor et al., 2010). Site-directed fluorescence labeling therefore involves mutating a specific residue of interest to a Cys residue by SDM and covalent attachment of a thiol-reactive extrinsic fluorophore. The choice of Cys is very attractive for site-specific labeling due to its low abundance throughout the proteome. An efficient and rapid procedure for cysteine labeling in proteins has been described (Kim et al., 2008). However, if the protein contains several cysteines, they need to be converted to a single cysteine mutant without perturbing the function. For instance, one of the earliest Cys scanning mutagenesis studies has produced functional lactose permease monomer devoid of eight native cysteine residues (Kaback and Wu, 1999). The use of site-directed biochemical and biophysical techniques has provided detailed mechanistic insights about the function of lactose permease especially in the absence of crystal structure. Recently, “cysteine metal protection and labeling” (CyMPL) method has been shown to be useful to specifically label the cysteine of interest in a protein mixture or in proteins containing several cysteines, or in the native environment of proteins (Puljung and Zagotta, 2011). This method involves reversible protection of the desired cysteine by binding metal ions and the background cysteines are blocked with nonfluorescent covalent modifiers with minimal perturbation of proteins. This method has the capability to specifically label proteins with multiple fluorophores in a controlled fashion.

### Extrinsic Fluorophores—NBD and Bimane as Ideal Fluorophores for Intramolecular Protein Motions and Conformational Dynamics

The biggest advantage offered by extrinsic fluorophores is the availability of plethora of fluorophores that are tailor-made for specific applications (Haugland, 2005; Hawe et al., 2008). Most extrinsic fluorescent labels display high sensitivity to environmental polarity. Further, these fluorophores are weakly fluorescent or non-fluorescent in water but fluoresce strongly upon binding to membranes and membrane peptides/proteins, which makes the contribution from the unbound probe negligible. There are few specific criteria for the choice of the extrinsic fluorophores for site-specific incorporation throughout the protein, which are as follows:

- The size of the fluorophore and linker (if any) should be small enough so as not to perturb the structure of the protein and can be efficiently inserted in relatively hydrophobic regions.
- The fluorophore should be sensitive to changes in environmental polarity, i.e., it should exhibit environment-sensitive fluorescence. This is an important criteria for monitoring the membrane binding events and topology of the protein (whether solvent or lipid exposed) etc. However, for monitoring the conformational kinetics of membrane proteins utilizing resonance energy transfer and distance measurements, the environment-insensitive fluorophores are usually preferred.
- Excitation and corresponding emission wavelengths should ideally be in the visible range so as to minimize fluorescence contributions from intrinsic Trp and Tyr residues.
- Since a single fluorophore per molecule is expected, the fluorophore should have a high quantum yield to get good signal-to-noise ratio.

**Figure 3:**
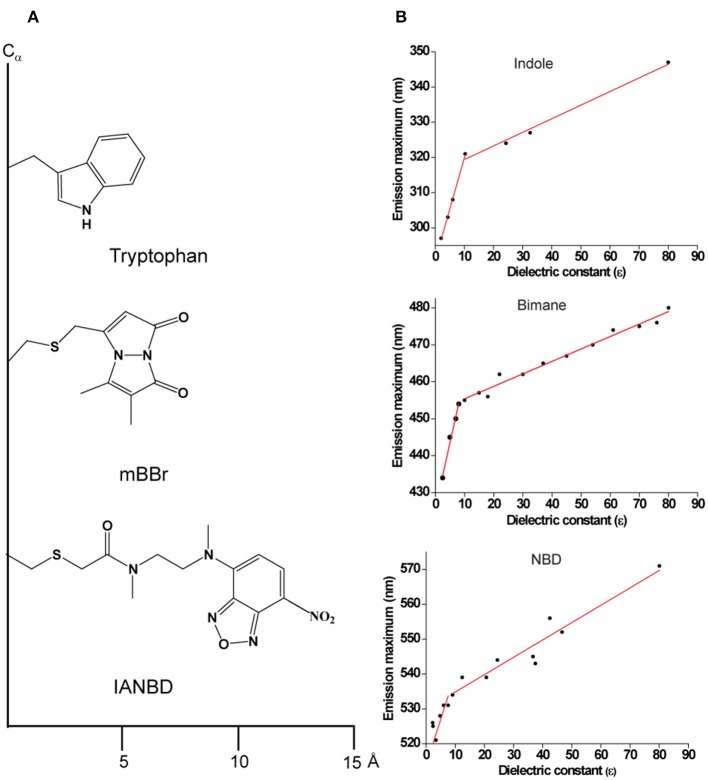
**(A)** Schematic representations of Tryptophan; monobromobimane (mBBr) and *N, N*′-Dimethyl-*N*-(iodoacetyl)-*N*′-(7-nitrobenz-2-oxa-1,3-diazol-4-yl) (IANBD) showing their size from the point of attachment to the C_α−_ protein backbone. **(B)** Environment-sensitivity of widely used fluorescent probes. A plot of emission maximum as a function of dielectric constant is shown for the widely used intrinsic fluorophore of a protein, Trp (indole as the fluorescing group) and for the popular extrinsic probes for site-directed fluorescence, NBD and bimane. Data are adapted from Sun and Song (1977) (indole); Fery-Forgues et al. (1993) (NBD); Ho et al. (2013) (bimane) and plotted. See text for details.

There are hundreds of extrinsic fluorescent probes, particularly several thiol-reactive probes, available commercially (Haugland, 2005). Despite the availability of a plethora of extrinsic fluorophores, only a few have been the most widely used in sequential site-specific incorporation to monitor the structural dynamics of membrane proteins. They are NBD, 7-nitrobenz-2-oxa-1,3-diazol-4-yl (Chattopadhyay, 1990), and bimane (Kosower et al., 1982), which meet all the criteria to serve as an excellent probe for spectroscopic and structural mapping of proteins (Figure 3). In aqueous medium, NBD is weakly fluorescent and fluoresces brightly in the visible range upon transfer to a hydrophobic medium and exhibits a high degree of environmental sensitivity (Chattopadhyay and London, 1988; Lin and Struve, 1991; Fery-Forgues et al., 1993; Chattopadhyay et al., 2002), which has been widely used in monitoring the orientation and conformational dynamics of membrane proteins (Crowley et al., 1993, 1994; Liao et al., 1997; Shepard et al., 1998; Shatursky et al., 1999; Raghuraman and Chattopadhyay, 2007b). Further, NBD-labeled lipids are well-established fluorescent analogs of native lipids to monitor membrane dynamics and various cellular activities (Kobayashi and Pagano, 1988; Wüstner et al., 2001; Mukherjee et al., 2004; Elvington et al., 2005; Raghuraman et al., 2007). Since NBD is a polar fluorophore and its dipole moment changes by ~4D upon excitation (Mukherjee et al., 1994), its fluorescence has been extensively utilized to investigate the organization and dynamics of membranes and hydration dynamics of membrane proteins (Chattopadhyay et al., 2002; Raghuraman and Chattopadhyay, 2007a; Raghuraman et al., 2014) utilizing the Red Edge Excitation Shift (REES) approach (see later). Importantly, due to visible range excitation and emission properties, NBD is also a suitable probe for microscopic and cell biological studies (Kobayashi and Pagano, 1988; Chattopadhyay, 1990; Elvington et al., 2005).

Like Trp and NBD, bimane is a small (comparable to the size of Trp), environment-sensitive probe (Figure 3) with well-characterized spectral properties (Kosower et al., 1982; Mansoor et al., 2010) and has been widely used to monitor the functionally-relevant dynamic structural changes in several membrane proteins (Islas and Zagotta, 2006; Yao et al., 2006; Semenova et al., 2009; Tsukamoto et al., 2009; Tsukamoto and Farrens, 2013). Further, there are several derivatives of bimane available that are quite useful for tryptophan-induced quenching (TrIQ) studies to monitor protein structure and dynamics (Mansoor et al., 2002, 2010; Mansoor and Farrens, 2004 and see later). These include monobromobimane (mBBr), a positively charged bimane (qBBr) and (2-pyridyl)dithiobimane (PDT-bimane). There are few disadvantages of using bimane for specific applications. Unlike NBD, bimane is a nonpolar fluorophore and is therefore not suitable for monitoring solvent relaxation/hydration dynamics. In addition, it has a low molar absorption coefficient (ϵ_380nm_ = 5,000 M^−1^cm^−1^) compared to NBD (ϵ_478nm_ = 25,000 M^−1^cm^−1^). Further, it is not a suitable probe for microscopic and cell-based biological applications since bimane requires UV excitation (~380 nm).

In addition to small extrinsic fluorescent dyes, several novel fluorescent reporters are also available for imaging in cell biology. In fact, a comprehensive “glowing fluorescent toolbox” is now available to assess protein localization and function utilizing microscopy-based sophisticated fluorescence spectroscopic approaches in mammalian systems (Giepmans et al., 2006; Rodriguez et al., 2017).

## Site-Directed Fluorescence (SDFL) Approaches

**Figure 4:**
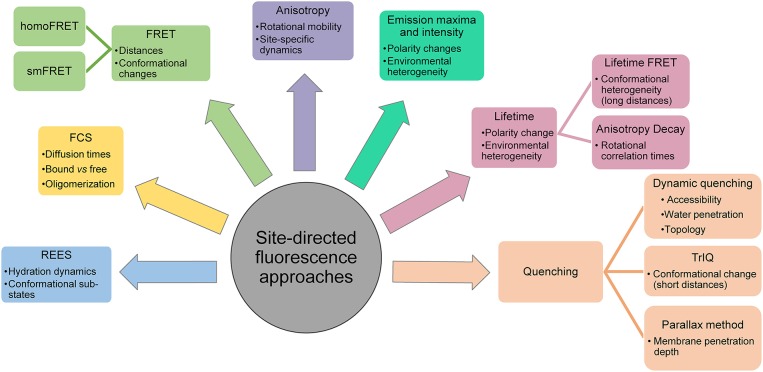
Applications of site-directed fluorescence approaches used in dynamic structural biology of membrane proteins.

In general, fluorescence provides several suitable parameters to obtain information regarding protein stability, folding, membrane binding, topology, conformational dynamics, solvent relaxation/hydration dynamics, lipid-protein interactions etc. These include the fluorescence emission maximum, steady-state fluorescence intensity, apparent quantum yield, mean fluorescence lifetime, fluorescence anisotropy (rotational correlation times), bimolecular quenching constant and energy transfer efficiency (Figure 4). In other words, an advantage of SDFL approaches is the multiplicity of measurable parameters which are complementary and can effectively be used to derive structural and dynamic information. SDFL is therefore useful in monitoring the structural dynamic changes along with simultaneous assessment of the protein function. Further, correlating spectral data with changes in structural and functional states of a protein often provides direct and unambiguous information about the mechanistic details associated with the function of proteins under physiological conditions making SDFL approaches a very powerful tool (Heuck and Johnson, 2002; Johnson, 2005). This is particularly true for membrane peptides and proteins for which there is no high-resolution structure available or when the structural information is limited. This is reflected in the extensive use of SDFL methods to study changes in conformational dynamics in different classes of membrane proteins like pore-forming peptides and proteins (Nagahama et al., 2002; Parker and Feil, 2005; Raghuraman and Chattopadhyay, 2007a; Haldar et al., 2008; Ho et al., 2013), GPCR (Yao et al., 2006; Daggett and Sakmar, 2011; Dekel et al., 2012; Alexiev and Farrens, 2014), potassium channels (Cha and Bezanilla, 1997, 1998; Cha et al., 1999; Raghuraman et al., 2014), inward-rectifying potassium channels (Wang et al., 2016, 2018, 2019), mechanosensitive ion channels (Wang et al., 2014; Martinac, 2017), ligand-gated ion channels (Sasmal and Lu, 2014), membrane transporters (Liu and Sharom, 1996; Verhalen et al., 2012; Terry et al., 2018), and intrinsically disordered proteins (Ferreon et al., 2009). Therefore, the wide applicability of SDFL approaches to study diverse systems makes fluorescence a sophisticated yet reliable technique for ensemble and single molecule measurements in both *in vitro* and *in vivo*. This review is focused on the SDFL approaches (see Figure 4) and their applications to explore the dynamic structural biology of membrane peptides/proteins.

### Fluorescence Emission Maximum and Fluorescence Intensity to Monitor Membrane Partitioning and Secondary Structure of Membrane Proteins

Pore-forming peptides and toxins can exist as stable water-soluble state as well as in membrane-bound state and the conversion between these two states usually involves large conformational changes (Parker and Feil, 2005; Christie et al., 2018). In many cases, the membrane binding promotes the formation of secondary structures (folding) in pore-forming toxins (Ladokhin and White, 1999). In addition, the membrane binding efficiency, orientation and pore-formation depend on the phase state and the lipid composition of membranes. Importantly, the partitioning of soluble proteins to membranes and their refolding within membranes are of significant importance to many physiological and disease processes. Furthermore, the high-resolution structures of the soluble forms of these proteins have been solved using X-ray crystallography or NMR, however, capturing the high-resolution snapshot of membrane-bound/inserted forms of these proteins still remains a formidable challenge (Christie et al., 2018). In this regard, steady-state fluorescence emission spectra and its associated fluorescence intensity can offer enormous insights into the membrane partitioning, orientation and dynamics of pore-forming peptides and toxins. The fluorescence emission spectrum reveals information about the polarity of the immediate environment around a fluorophore (Chattopadhyay et al., 2002; Raghuraman and Chattopadhyay, 2003; Demchenko et al., 2009). Specifically, when an environment-sensitive fluorophore changes its location from polar (aqueous) to a non-polar (membrane) environment, the emission spectrum generally increases in fluorescence intensity with a concomitant shift in the fluorescence emission maximum toward shorter wavelengths (blueshift). Thus, following these changes can monitor the partitioning of peptides/proteins between the aqueous solution and membranes (Raghuraman and Chattopadhyay, 2004c; Koehorst et al., 2008; Ho et al., 2013; Kyrychenko and Ladokhin, 2018).

The intrinsic Trp fluorescence is a widely used classical tool to investigate membrane binding events of proteins. For instance, the presence of cholesterol has been shown to reduce the binding of the pore-forming hemolytic honey bee venom peptide, melittin, to membranes utilizing the intrinsic Trp fluorescence (Raghuraman and Chattopadhyay, 2004c). Because melittin at low concentration adopts random coil conformation in buffer, its single Trp residue exhibits an emission maximum at 352 nm. Upon binding to membrane interface, melittin adopts α-helical conformation (Raghuraman and Chattopadhyay, 2004b,c) and exhibits emission maximum of 336 nm. The melittin-membrane binding has therefore been quantitated by the blue shift (from 352 to 336 nm) of fluorescence emission maximum of the single Trp residue of melittin upon binding to liposomes. In this case, the ratio of fluorescence intensities monitored at 336 nm (membrane-bound) and 352 nm (in buffer) has been effectively used to derive the binding curves of melittin with membranes containing different amounts of cholesterol (Raghuraman and Chattopadhyay, 2004c). Similar use of the ratio of fluorescence intensities has been used to monitor the membrane binding of the native as well as scrambled N-domain peptide (34-mer) of the chemokine receptor CXCR1, a peptide-binding GPCR (Haldar et al., 2010b). However, the fluorescence intensities ratio may not a reliable parameter when it comes to evaluating protein unfolding transitions because it leads to incorrect determination of thermodynamic parameters (Zoldak et al., 2017).

Further, increased fluorescence intensity concomitant with blue shifted emission maximum has been observed during membrane binding events in many cases like C-domain of the *Clostridium perfringens* alpha-toxin using the site-directed fluorescence of Acrylodan and NBD (Nagahama et al., 2002), pH-dependent association of the apoptotic repressor Bcl-xL with membranes utilizing NBD fluorescence (Vargas-Uribe et al., 2013; Kyrychenko and Ladokhin, 2018), and protonation-trigged membrane interaction of diphtheria toxin T-domain (Perier et al., 2007). Pore formation by equinatoxin II, an eukaryotic pore-forming toxin representative from sea anemones, involves a novel single helix insertion mechanism has been shown using site-directed NBD fluorescence (Malovrh et al., 2003). The number of LukF (leucocidin fast fraction) components of Staphylococcal γ-hemolysin has been calculated in a single pore utilizing changes in single molecule Badan fluorescence (Nguyen et al., 2006).

One of the earliest studies that utilized SDFL approaches to probe conformational changes in an ion channel has been carried out on the voltage-dependent *Shaker* K^+^ channel (Mannuzzu et al., 1996; Cha and Bezanilla, 1997, 1998; Glauner et al., 1999). Particularly, the spectroscopic mapping of the voltage sensor (S4 helix) movement in *Shaker* K^+^ channel has been done using a combination of electrophysiology measurements and SDFL (called “voltage-clamp fluorometry”) to monitor the voltage sensor movement during voltage-dependent gating. This combined approach of using electrophysiology and fluorescence measurements gives an opportunity to simultaneously measure both the function and functionally-relevant structural changes using tetramethylrhodamine (TMR) fluorescence (Glauner et al., 1999). These changes correlated with gating, activation, and slow inactivation of the channel (Zheng and Zagotta, 2003). Change in fluorescence intensity measurements often give useful insights about the nature of conformational changes in a protein even though it cannot map the magnitude or direction of a movement of protein that undergoes functionally associated structural changes. For instance, as stated above, blue shifts in fluorescence emission maximum indicate a change in location of a labeled residue to a more nonpolar/hydrophobic environment. Further, if a set of labeled sites shows significantly large and correlated changes, this might indicate that the region of protein that contains the labeled residues experiences the conformational change in a coordinated manner. The resting state of a K^+^ channel has been modeled using this type of analysis (Pathak et al., 2007). Other membrane proteins such as KcsA K^+^ channel (Blunck et al., 2008), Na_V_ channels (Chanda et al., 2004), Hv1 channels (Tombola et al., 2010), Ci-VSP (Villalba-Galea et al., 2009; Kohout et al., 2010), CNG channels (Zheng and Zagotta, 2000), and glutamate transporters (Larsson et al., 2004) have been mapped with intensity measurements coupled with functional manipulation to study the mechanism of protein rearrangement.

It is important to note that the magnitude of fluorescence changes is not always proportional to the magnitude and direction of conformational changes due to complex nature of fluorescent processes in heterogeneous environment (Taraska, 2012). This is especially true if only one labeled site is used. Sequential labeling of Cys residues with NBD or bimane and observing the fluorescence changes can yield secondary structural details in solution and membranes. The secondary structural elements have been predicted from the observed bimane fluorescence parameters (Musse et al., 2006; Wei et al., 2007; Ho et al., 2013) using the method of harmonic wave function analysis (Cornette et al., 1987). This method works because the motion of the protein-attached fluorescent label will be confined to certain defined angle and therefore the electronic center of the attached label will fluctuate in “wobble in a cone” manner. This probably makes the attached label to experience an average properties of local environment with respect to refractive index, dielectric constant, electric field etc. during its excited-state lifetime. As a result, for a helix that is labeled in all residues, the measured fluorescent property of respective probes will follow a change in amplitude and angular frequency as described by a harmonic wave function. This has been shown in case of colicin E1, which forms voltage-dependent ion channels in *E. coli* cells. Site-directed bimane fluorescence has been used demonstrate that helix 1 and helix 3 of the membrane-associated closed state of the channel is amphipathic α-helix oriented parallel to the membrane surface (Musse et al., 2006; Wei et al., 2007). In addition, a new model for the membrane-associated colicin E1 ion channel in the closed state has been developed using site-directed bimane fluorescence and novel helical periodicity analysis method (Ho et al., 2013). The above-mentioned secondary (helical) structure prediction in membrane proteins by fitting the data of measured fluorescence parameters with a harmonic wave function (Musse et al., 2006) has also been exploited to monitor the conformational changes in the translocon channel structure in an intact, functional membrane-embedded complex (Alder et al., 2008).

In another example, sequential site-directed NBD labeling has been used to monitor the membrane-induced secondary structural changes in cytolytic toxins. Many pathogenic gram-positive bacteria produce cholesterol-dependent cytolysins (CDC) (formerly “thiol-activated” cytolysins). The secreted forms of toxin exist as highly water-soluble monomers, yet they form large homooligomeric compelexes in the membrane—upto fifty individual monomers for perfringolysin O (Olofsson et al., 1993; Christie et al., 2018). As the name suggests, the cytolytic action of CDCs critically requires the presence of cholesterol in membranes. Several excellent studies examining the periodicity of site-directed NBD fluorescence in the labeled perfringolysin O have identified the membrane-spanning domain of this toxin and how membrane insertion promotes the structural transition upon membrane binding. Importantly, novel mechanistic insights of membrane insertion for a CDC have come through site-directed fluorescence approaches (Shepard et al., 1998; Shatursky et al., 1999; Ramachandran et al., 2004; Johnson, 2005; Christie et al., 2018).

Since transmembrane α-helices usually extend beyond the lipid bilayer and several high-resolution structures of membrane proteins have only a few lipids, defining the membrane boundary for these helices is therefore challenging. Site-specific NBD labeling and fluorescence has been shown to be a powerful tool to identify the ends of transmembrane α-helices in the bacterial diacylglycerol kinase (Jittikoon et al., 2007), which is the smallest known kinase. Further, fluorescence measurements also reveal that the hydrophobic matching between the hydrophobic core of the membrane and the diacylglycerol kinase is highly efficient for a significant variation in the bilayer thickness. In fact, the environment sensitivity of tryptophan has also been shown to be useful in defining the ends of transmembrane helices as in the case of mechanosensitive channel of large conductance, MscL (Powl et al., 2005). These studies clearly highlight the potential of such site-directed fluorescence approaches in dynamic structural biology of membrane proteins.

### Fluorescence Polarization/Anisotropy to Monitor Membrane Partitioning and Protein Motions

Protein motions, particularly their conformational dynamics, regulate the function of proteins (Henzler-Wildman and Kern, 2007). Polarization or anisotropy studies have been used to study protein interactions for more than 50 years and is still a valuable tool to quantify of interaction of proteins in micro- and nanomolar concentration (Jameson and Ross, 2010; James and Jameson, 2014). Since fluorescence polarization provides information on molecular flexibility and rotational motion, it is also a powerful tool to monitor the structural dynamics of soluble and membrane proteins/peptides. Because the dynamics of the fluorescent dye is influenced by the motion of its attached region in a protein, valuable information regarding protein structure, dynamics and conformational changes can be obtained by fluorescence anisotropy or fluorescence polarization measurements. Molecules that are freely mobile (i.e., on the surface of a protein) will display a low anisotropy. However, molecules that are membrane-bound or at buried sites exhibit a higher anisotropy due to hindered rotational mobility of the probe. Membrane binding of melittin and how cholesterol influences its partitioning to membranes have been studied using the polarization of Trp fluorescence (Raghuraman and Chattopadhyay, 2004c). In the pH-gated potassium channel KcsA (Cuello et al., 2010; Raghuraman et al., 2012, 2014; Kratochvil et al., 2016), mapping the differences in the steady-state NBD polarization values between the inactivated and non-inactivated/conductive conformations has demonstrated the gating-induced differences in rotational dynamics of KcsA outervestibule (Raghuraman et al., 2014). Interestingly, the functional-state dependent changes in local dynamics of outervestibule in KcsA, detected by site-directed NBD fluorescence, have not been captured in the electron paramagnetic resonance (EPR) time scale. The reason could be the differences in timescales of fluorescence (ps-ns) and EPR (ns-μs) sensitivity to protein motions (Thomas, 1978; Alexiev et al., 2003). Since the outervestibule of KcsA not properly structured, the observed dynamic changes between the functional states using fluorescence approaches are probably averaged out in the time scale of EPR sensitivity (Raghuraman et al., 2014). Considering the motion of the lower activation gate of KcsA is sensitive to EPR time scale (Perozo et al., 2002), these results reinforces the existence of heterogenous dynamic modes throughout the protein.

It should be noted that the excited-state fluorescence lifetime of a probe strongly influences the steady-state anisotropy/polarization values (Lakowicz, 2006). Therefore, care must be taken to ensure that the changes in anisotropy/polarization values are not due to lifetime changes, which can be easily achieved by calculating the apparent rotational correlation times using Perrin's equation (Lakowicz, 2006) with the knowledge of limiting anisotropy (r_o_) of the fluorophore. For instance, r_o_ value for Trp is 0.16 (Eftink et al., 1990) and the corresponding value for NBD is 0.354 (Mukherjee et al., 2004). While steady-state polarization (anisotropy) provides information about the overall dynamics of the protein, time-resolved anisotropy measurements are useful in isolating the local, segmental and global motions. Since fluorescent dyes in complex heterogenous systems generally have many lifetime components due to various dynamic modes like global motion (tumbling of whole protein with attached probe), segmental motion (probe mobility mediated by protein's local dynamics), and local motion (rotational motion of tethered probe with respect to attached protein), rotational correlation times (τ_*c*_) are calculated to resolve the various dynamic modes in their timescale (Krishnamoorthy, 2018b).

Time-resolved anisotropy studies have been used on the AB-loop, EF-loop of bacteriorhodopsin (bR) and the fourth cytoplasmic loop of bovine rhodopsin to monitor the rotational dynamics and conformational changes in the inactive state (Alexiev et al., 2003; Schroder et al., 2005; Alexiev and Farrens, 2014). The conformational changes of the loop have been identified by assigning the τ*c* to the loop. In another study, time-resolved fluorescence anisotropy experiments in a fluorescein-bound cation channel channelrhodopsin-2 (CrChR2) have been used to monitor the light-induced conformational dynamic changes near the inner gate in closed and open states of the channel (Volz et al., 2016). The observation of increased fluorescence anisotropy for the light-induced prolonged conducting state of fluorescein-bound CrChR2 evidently suggests that large conformational changes, particularly the outward tilt of helix B, is coupled with the transition to open state at the cytoplasmic surface. Since anisotropy/polarization quantifies the extent of change in orientationally-distributed emitting states after initial photoselection, the emission anisotropy/polarization is not only influenced by change in lifetimes and protein motions, but also due to homo-FRET. Hence, the influence of homo-FRET in fluorescence anisotropy decay can potentially be used to monitor membrane protein oligomerization (Clayton, 2018).

### Fluorescence Lifetimes

Fluorescence emission intensity changes may not always be a reliable parameter to monitor the location of probes due to its composite property that is dependent on several factors (Shepard et al., 1998; Turconi et al., 2001; Lakowicz, 2006; Zoldak et al., 2017). The fluorescence lifetime is an intrinsic property of the probe and therefore is an excellent indicator of fluorophore's local environment (Prendergast, 1991; Berezin and Achilefu, 2010). When a fluorophore is excited, every dye molecule stays in the excited state briefly (typically nanoseconds) and relaxes back to the ground state with an emission. The dwell time period in the excited state is called the fluorescence lifetime (τ), which is the reciprocal of the sum of all transition rates back to the ground state (Lakowicz, 2006). In general, the emission lifetimes of Trp, NBD and other flurorophores are higher in nonaqueous than aqueous environment. Particularly, the fluorescence lifetime of NBD is highly sensitive to its local environment (Lin and Struve, 1991; Chattopadhyay et al., 2002) and the magnitude of lifetime directly reveals the environment of the probe and in particular its exposure to water (Crowley et al., 1993). NBD has a short lifetime of ~1.5 ns in aqueous media and has longer lifetimes (~5-10 ns) in a nonpolar milieu (Fery-Forgues et al., 1993; Chattopadhyay et al., 2002; Johnson, 2005; Raghuraman et al., 2007). Hence, measurements of fluorescence lifetime can distinguish the fluorophore's location as well as the heterogeneity of probe locations if more than one lifetime is observed in a given sample. As a result, lifetime distribution analyses can provide an ultrafast snapshot of the population distribution of fluorophores (Krishnamoorthy, 2018a). Site-directed NBD fluorescence lifetimes have been extensively utilized to characterize the orientation of melittin in membranes containing varying lipid composition (Raghuraman and Chattopadhyay, 2007b), cholesterol-dependent cytolysins with respect to membrane partitioning regions, structural transitions during insertion and pore formation (Shepard et al., 1998; Shatursky et al., 1999; Heuck et al., 2000; Ramachandran et al., 2004), movement of NBD-Lys signal sequence of a nascent protein in endoplasmic reticulum membrane (Crowley et al., 1993), and cotranslational integration of eukaryotic multispanning polytopic membrane proteins (Lin et al., 2011). Frequency-domain fluorescence lifetime measurements on tetramethylrhodamine-labeled KcsA mutants at the helix bundle crossing at the lower activation gate have revealed two channel populations with different lifetimes. Interestingly, the relative distribution of these lifetimes has been shown to be in excellent agreement with the open probability (i.e., the function) of KcsA K^+^ channel. Comparison of lifetimes obtained in wildtype KcsA, which undergoes C-type inactivation and has low open probability, and mutant channels that has increased open probability, it has been demonstrated that the selectivity filter of KcsA potassium channel acts as the crucial second gate for ion conduction (Blunck et al., 2006).

Generally, fluorescence decays are fitted with a sum of a few (1–4) discrete exponentials to obtain mean lifetimes in a given system. Apart from fitting the fluorescence decay curves with discrete exponentials, the mean fluorescence lifetime can also be obtained in a model-independent manner from the histogram of photons counted during the measurement as recently described (Fiserova and Kubala, 2012; Chatterjee et al., 2019). However, when a fluorophore is attached to a protein, its excited state lifetime population is a distribution of various degrees of heterogeneity (Krishnamoorthy, 2018a). Therefore, fitting the fluorescence intensity decay data to a probability distribution of lifetimes is judicious than conventional fitting of fluorescence decay curves to a sum of discrete exponentials. This model-independent, probability distribution analysis is known as Maximum Entropy Method (MEM), which maximizes the entropy while minimizing the χ^2^ has been widely used for analysing fluorescence lifetime distribution (Brochon, 1994; Esposito et al., 2015; Smith et al., 2017; Krishnamoorthy, 2018a).

In Na,K-ATPase, which contains 16 Trp residues, MEM analysis of tryptophan lifetimes has shown that long-living excited state population of Trp is heterogeneous, and the lifetime distribution is emission wavelength dependent (Demchenko et al., 1998). This underlines the significantly greater emission heterogeneity of multi-tryptophan containing proteins when compared to single-tryptophan proteins. Another multi-tryptophan containing protein in which MEM has been used to reveal the conformational heterogeneity is the gramicidin ion channel. Gramicidin channels can be stabilized in channel and non-channel conformations, whose architecture is dramatically different in both these states (Rawat et al., 2004). The four Trp residues in gramicidin channel conformation forms the “aromatic ring” in the interfacial region of the membrane, however, in the non-channel conformation, the Trp residues are spread across the protein along the membrane axis. MEM analysis in membrane-bound gramicidin ion channels has shown that the Trp residues in the non-channel state encounters relatively heterogeneous environment compared to the channel conformation. These results support the general opinion that structured proteins exhibit sharp distributions of Trp lifetimes, which is in contrast to relatively broad distributions obtained with unfolded/denatured proteins (Rawat et al., 2004; Haldar et al., 2010a). Although MEM analysis of fluorescence lifetimes is a well-developed analysis tool for monitoring heterogeneous organization and dynamics of native membranes (Mukherjee et al., 2007a) and folding of small soluble proteins (Lakshmikanth et al., 2001; Jha et al., 2009), it is under-utilized in monitoring the functionally-relevant conformational heterogeneity of membrane proteins.

### Application of Fluorescence Quenching to Membrane Proteins

Fluorescence quenching results in decreased fluorescence emission intensity when there is an interaction between a fluorescent dye and a quencher (usually another molecule or a group). The interaction of quencher with the fluorescent dye in its excited state facilitates the deactivation rates of the excited state of the fluorescent dye. Therefore, the degree of quenching is governed by the quencher concentration that determines the close proximity with the fluorescent dye and also the competition between fluorescence and all the deactivation processes. In general, the quenching mechanism is of two major types depending on the motions of fluorophore and quencher typically in nanosecond time scale: dynamic (collisional) and static quenching (Eftink, 1991; Chattopadhyay and Raghuraman, 2004; Lakowicz, 2006). In bulk, non-viscous media, the distance between fluorophore and quencher molecules rapidly (dynamically) change and the quenching phenomenon happens only at times when close encounters of fluorophores and quenchers happen. This “dynamic quenching” is also called “collisional quenching” when the quenching of fluorescence emission intensity occurs due to only collisions between fluorescent dye and the quencher. On the other hand, the quenching mechanism is called “static quenching” when the distances between quencher and the fluorescent dye do not significantly change within the lifetime of the dye. This type of quenching is common in frozen or highly viscous solutions and membranes. The membrane components (lipids and proteins) laterally diffuse very slowly in membranes with a diffusion coefficient in the range of 10^−8^-10^−12^ cm^2^sec^−1^.

#### Collisional Quenching Using Aqueous Quenchers

Since the magnitude of fluorescence quenching depends on the accessibility of the fluorescent dye to the quencher, dynamic (collisional) quenching using the widely used neutral (acrylamide) and anionic (iodide ions) quenchers has been extensively utilized to explore the topology (surface exposed, buried or membrane-bound) of Trp residues in soluble and membrane proteins (Lehrer, 1971; Eftink, 1991; Raghuraman and Chattopadhyay, 2004a,b,c). For a better quantitative understanding of quenching, the fluorescence intensity vs. quencher concentration is plotted. The slope of such a plot termed the Stern-Volmer constant (K_SV_) can give an idea of the degree of accessibility of the fluorophore, i.e., high values of slope means the increased extent of exposure to quencher. It is well known that the K_SV_ value for a completely exposed Trp is ~18 M^−1^, whereas it is in the range of ~4–7 M^−1^ for Trp localized at the micellar interfacial region (Raghuraman and Chattopadhyay, 2004a; Chatterjee et al., 2019), and the K_SV_ values of tryptophan localized at the interfacial region of the lipid bilayer is as low as ~2 M^−1^ (Raghuraman and Chattopadhyay, 2004b,c). However, there is a caveat while comparing results between samples only on K_SV_ values, because these values are only relevant if the fluorescence lifetime does not change significantly between the samples. For this reason, the bimolecular quenching constant (k_q_) should be used over K_SV_ as the former corrects for differences in fluorescence lifetimes in the absence of quencher (Johnson, 2005; Lakowicz, 2006).

Two of the most popular efficient aqueous quenchers of NBD fluorescence are cobaltous (Homan and Eisenberg, 1985; Morris et al., 1985; Chattopadhyay and London, 1988; Raghuraman et al., 2004; Raghuraman and Chattopadhyay, 2007b) and iodide ions (Crowley et al., 1993; Shepard et al., 1998; Shatursky et al., 1999; Heuck and Johnson, 2002; Lin et al., 2011). Since both cobalt and iodide ions are charged, they are soluble in water and do not pass through the hydrophobic core of the membrane and the quenching of NBD fluorescence by them give us information about the insertion mechanism, topology and orientation of membrane-bound peptides and proteins. For instance, if a residue is particularly exposed to the aqueous environment it would show a higher k_q_ compared to a labeled residue moving to a nonpolar environment, which could be either protein interior or moving into hydrophobic core of the membrane. In both scenarios, low values of k_q_ is obtained and even lifetime measurements will not show significant differences because the overall microenvironment of the probe is nonpolar in nature. One can use additional quenching measurements in which the membrane is doped with nitroxide labeled phospholipids (efficient quencher of any fluorescence) to distinguish between the above scenarios. Cobalt quenching of NBD has been previously used to understand the effect of cholesterol and anionic lipid, dioleoylphosphatidylglycerol (DOPG) in changing the orientation of membrane-bound melittin (Raghuraman and Chattopadhyay, 2007b). Bacterial toxins punch holes in membranes by either inserting as α-helices or β-barrels and this insertion mechanism has been studied in detail using iodide quenching of NBD-labeled pore-forming toxins like cholesterol-dependent cytolysins and Bax (Shepard et al., 1998; Shatursky et al., 1999; Johnson, 2005; Kale et al., 2014). The pattern of NBD exposure of NBD-labeled residues of a transmembrane segment (TMS) or an amphipathic sequence to the membrane can be obtained by nitroxide labeled phospholipid quenching. For instance, a typical β-hairpin will show alternating aqueous and nonaqueous environment variation and the quenching pattern can also be subjected to helical wheel analysis to obtain the helical nature of the peptide or protein (Heuck and Johnson, 2002).

#### Parallax Method and Distribution Analysis to Monitor Penetration Depths of Membrane-Bound Peptides and Proteins

“Membrane penetration depth” is a valuable parameter in the study of structural organization of membranes and topology, orientation and folding membrane-bound peptides/proteins. This is because the lipid bilayer has both mobility and polarity gradients along its axis and therefore properties like water penetration, polarity, hydrogen bond forming capability, segmental dynamics change dramatically in a depth-dependent manner in membranes. An important application of fluorescence quenching has been to quantitatively characterize the membrane penetration depths of membrane-bound proteins/peptides by using the quenchers (usually nitroxide spin labels or heavy atoms like bromine) that are covalently attached to the polar/hydrophilic headgroup or to a specific regions of acyl chains of phospholipids which gives the quencher a defined depth (London and Ladokhin, 2002). Here, the interactions of fluorophore (either membrane-embedded or protein-labeled) involve predominantly static quenching within a typical range of 8–12 Å distance between the fluorophore and the quencher (Chattopadhyay and London, 1987; Ladokhin, 1999). Generally, the quenchers are distributed in both leaflets of the lipid bilayer to account for the trans-leaflet quenching of highly penetrated proteins or peptides. There are two popular methods of depth analysis namely the parallax approach and distribution analysis (London and Ladokhin, 2002). In parallax method (Chattopadhyay and London, 1987), the apparent location of fluorescent label along the membrane axis is determined by using phospholipid quenchers at different depths and the best pair of quenchers is used in the membrane depth analysis. The advantage of this method is that it can provide the depth of the protein or peptide in angstrom resolution. This quenching approach has been widely used to characterize the membrane penetration depths of intrinsic Trp residues and other extrinsic probes labeled at specific sites in nicotinic acetylcholine receptor (Chattopadhyay and McNamee, 1991), hemolytic peptide melittin (Ghosh et al., 1997; Raghuraman and Chattopadhyay, 2004c, 2007b; Haldar et al., 2008), cholesterol oxidase (Chen et al., 2000), ricin (Ramalingam et al., 1994), calcium dependent membrane binding protein annexins (Meers, 1990), model ion channel peptide (Chung et al., 1992) and colicin (Palmer and Merrill, 1994). The parallax method has also been used to monitor the depths of penetration of NBD lipids and Nile Red in membranes (Mukherjee et al., 2004, 2007b). The distribution analysis method is similar to parallax method in many ways. However, this method involves fitting the fluorescence quenching data obtained from quenchers that are positioned at known depths in the membrane with a Gaussian function (Ladokhin, 1997, 2014; London and Ladokhin, 2002). This method has been used to monitor the depths of penetration of Trp in outer membrane protein Omp A (London and Ladokhin, 2002) and recently the depth of NBD-labeled mutants of diphtheria toxin T-domain (Kyrychenko et al., 2018).

#### Dual Quencher Analysis

“Dual fluorescence quenching” utilizes the combination of lipophilic and aqueous quenchers to calculate the quenching ratio (Q-ratio) to determine the topography of transmembrane helices of membrane proteins in model membranes (Caputo and London, 2003). The original method permits determination of membrane penetration depths of Trp residues in membranes by using two quenchers, acrylamide and 10-doxylnonadecane (10-DN), that are not located at fixed depths. The Q-ratio by these quenchers has been found to have a linear dependence on Trp depth in membranes and this quenching method can even be applied to situations in which the thickness of the lipid bilayer is changed. In addition to acrylamide, KI has also been used as an aqueous quencher in this method to determine the Q-ratio (Wei et al., 2007). In principle, residues that are surface-exposed in the membrane-bound state are quenched more efficiently by the aqueous quencher, which results in higher values of [(F_o_/F_aq_) – 1] where F_o_ and F_aq_ represents the fluorescence intensity of the fluorophore in the absence and presence of aqueous quencher, respectively. In contrast, if the residues buried in membrane-bound state are quenched more efficiently by the lipophilic quencher, it results in higher values of [(F_o_/F_lp_) – 1] where F_o_ and F_lp_ represents the fluorescence intensity of the fluorophore in the absence and presence of lipophilic quencher, respectively. Therefore, the quenching ratio (Q-ratio) of [(F_o_/F_lp_) – 1/ (F_o_/F_aq_) – 1] would give the relative penetration depth of membrane peptides/proteins (Caputo and London, 2003). This “dual fluorescence quenching” approach has been used successfully to determine the hydrophobic α-helix locations in membranes by measuring the Trp depth within the membrane. Further, in conjunction with SDFL, dual quencher analysis has been used to reveal the topology of helices 1, 3, 6, and 7 in colicin E1 channel (Musse et al., 2006; Wei et al., 2007; Ho et al., 2013) and the transmembrane helices of bacterial diacylglycerol kinase (Jittikoon et al., 2007).

#### Tryptophan-Induced Quenching (TrIQ) to Monitor Small-Scale Conformational Changes

As discussed above, quenching coupled with SDFL is a powerful tool to observe conformational changes in membrane proteins. Another important application of SDFL is measuring Förster resonance energy transfer (FRET). However, FRET studies are not suitable for small intra or inter-protein structural changes especially to determine how a secondary structure packs to form a tertiary structure. The reason being that most FRET pairs are relatively large, a 100% labeling efficiency is generally needed for the acceptor dye, and importantly, most of the FRET methods are suitable for determining long distances (~20–100 Å). Farrens lab has developed a relatively novel method namely the Tryptophan-Induced Quenching (TrIQ), which exploits the phenomenon of tryptophan to quench the emission intensity of some fluorophores by an energy transfer technique called photo-induced electron transfer (PET) (Mansoor et al., 2002, 2010; Callis, 2014). Generally, FRET and PET are two mechanisms, which leads to changes in fluorescence emission of a fluorophore due to the presence of a quencher in a distance-dependent manner. Unlike FRET, PET between organic fluorophores and suitable electron donating moiety (such as Trp) requires van der Waals contact for efficient quenching (Doose et al., 2009). This PET based quenching can therefore be used as a reporter for monitoring conformational dynamics in proteins, particularly, to study short range interactions of ~5–15 Å between Trp and the fluorophore labeled site in question. Like tryptophan, it has recently been shown that bimane fluorescence is also quenched by tyrosine in a distance-dependent manner and can therefore be utilized to map distances in proteins (Brunette and Farrens, 2014). The TrIQ method has several advantages associated with it: only one probe is used in a TrIQ study, the labeling efficiency need not be 100% as TrIQ monitors the fluorescence of the fluorophore in the continuous presence of “quencher” Trp, and the need for only low sample concentration. A popular acceptor probe for the TriQ approach is bimane, which is small enough (~10 Å) and can be easily labeled in inaccessible regions without perturbing the tertiary packing of the protein. The TriQ-bimane approach has been widely used to study conformational changes in GPCRs like β-adrenergic receptors (Yao et al., 2006; Tsukamoto et al., 2009). This approach has also been used to study the interaction of rhodopsin with transducin (Janz and Farrens, 2004), the dynamic variations in the structure of cyclic nucleotide-gated ion channel (Islas and Zagotta, 2006), the secondary structure near the S3–S4 loop of BK channel (Semenova et al., 2009). However, the study of TrIQ is not only limited to Trp-bimane pair, but also for other fluorophores like BODIPY (boron-dipyrromethene), lucifer yellow, and Atto-655, whose unique properties can be exploited to obtain comparatively larger conformational changes to study protein-protein interactions, drug screening assays etc. (Mansoor et al., 2010; Bohuszewicz and Low, 2018).

### REES Approach to Monitor Hydration Dynamics and Protein Conformational Substates

When the mechanistic details of the function of proteins and other organized molecular assemblies are proposed, the hydration dynamics is largely ignored because most of the fluorescence-based approaches provide information on fluorophore itself. However, it is known that there exists an intrinsic relation between the dynamics of water molecules (i.e., hydration dynamics) and protein fluctuations (Li et al., 2007). Further, hydration dynamics has been shown to play a crucial modulatory role in lipid-protein interactions (Raghuraman and Chattopadhyay, 2007b; Raghuraman et al., 2014) and ion channel selectivity (Noskov and Roux, 2007). Red edge excitation shift (REES) is a wavelength-selective fluorescence approach, which is a convenient tool to probe relative hydration dynamics and the environment-induced restriction and dynamics around organized molecular assemblies like membranes, proteins etc. REES is operationally defined as “the shift in the wavelength of maximum fluorescence emission toward higher wavelengths, caused by a shift in the excitation wavelength toward the red edge of the absorption band.” There are several excellent reviews written on this topic highlighting the genesis of REES, criteria for obtaining REES and its applications to study dynamic organization of membranes and structural insights of soluble and membrane proteins (Demchenko, 2002, 2008; Raghuraman et al., 2003, 2005; Chattopadhyay and Haldar, 2014).

Basically, when a polar fluorophore (like NBD) is placed in bulk non-viscous solvent, the reorientation of solvent (water in biological systems) molecules occurs at a picosecond time scale, so that complete reorientation of all solvent molecules around the excited state dipole of the polar fluorophore takes place within its excited lifetime (usually nanoseconds). Therefore, irrespective of the excitation wavelength used (465 to 515 nm in case of NBD), fluorescence emission maximum of NBD is invariant because the emission is observed only from the solvent-relaxed state and hence no REES will be observed (i.e., 0 nm REES). However, if the same probe is located in a motionally-restricted environments (viscous medium, membranes and membrane proteins etc.), the process of reorientation of solvent molecules is slowed down to nanoseconds or longer. Hence, changing the excitation wavelength from 465 to 515 nm shifts the emission wavelength toward longer wavelengths (see Demchenko, 2002, 2008; Raghuraman et al., 2003, 2005 for details). This gives rise to different magnitude of REES and indicates a restricted mobility of the surrounding environment with respect to the fluorescent probe. This has an application to probe conformational heterogeneity in proteins (see below).

Since REES offers knowledge on water reorientation dynamics, this approach is sensitive to variations in local hydration dynamics (Chattopadhyay et al., 2002; Raghuraman and Chattopadhyay, 2003). This has been widely exploited to investigate the dynamic organization of model membranes with physiologically-relevant lipid composition (Arora et al., 2004; Mukherjee et al., 2004, 2007b; Mukherjee and Chattopadhyay, 2005) and native hippocampal membranes (Mukherjee et al., 2006) and, lipid-protein interactions (Raghuraman and Chattopadhyay, 2004a,b,c; Raghuraman et al., 2004, 2006, 2007; Rawat et al., 2004; Haldar et al., 2010b), folding, topology and hydration dynamics of soluble and membrane proteins (Tory and Merrill, 2002; Rawat et al., 2004; Raghuraman and Chattopadhyay, 2006; Raghuraman et al., 2014; Mishra and Jha, 2019). Using site-directed NBD fluorescence and REES approach, it has recently been shown that significant differences exist in hydration dynamics when the pH-gated potassium ion channel KcsA (Cuello et al., 2010; Raghuraman et al., 2012; Kratochvil et al., 2016) shuttles between open/conductive to open/inactivated conformation (Raghuraman et al., 2014). Precisely, the inactivated conformation of KcsA is correlated with the presence of restricted/bound water molecules in the outervestibule, whereas the open/conductive state has a relatively fast solvent relaxation. The increased hydration dynamics has been found to be linked with the highly dynamic outervestibule of KcsA in open/conductive conformation. This study supports the role of differential water dynamics in different functional states of KcsA and the idea that water could act as a structural component in selectivity filter gating mechanisms of potassium ion channels (Ostmeyer et al., 2013; Raghuraman et al., 2014).

Until recently, the application of REES approach is mainly restricted to obtain qualitative information related to hydration dynamics using the magnitude of REES in soluble and membrane proteins (Raghuraman et al., 2005, 2014; Chattopadhyay and Haldar, 2014). A novel application of REES approach has recently been reported, which demonstrates that the REES approach can be used as a powerful tool to probe dynamic proteins with a broad equilibrium of conformational states (Catici et al., 2016). Until recently, the potential of REES as a quantitative probe of molecular heterogeneity has been reported in the isolated voltage sensor of a voltage-gated K^+^ channel, KvAP. The results, which have been obtained using this novel approach of REES for membrane proteins for the first time, demonstrate that the physiologically-relevant paddle motif loop of the voltage sensor has lesser number of discrete equilibrium conformational states in phospholipid membranes compared to its organization in detergent micelles (Das et al., 2019).

### Fluorescence Correlation Spectroscopy

Biological systems are complex and intrinsically heterogeneous with variable distributions of structural conformations. Analysis of these distributions can be useful in deciphering the mechanism of action of biological systems (Johnson et al., 2005; Garcia-Saez and Schwille, 2007). The individual inhomogeneities in the biological molecule can be studied in detail utilizing single-molecule techniques compared to ensemble measurements (Haustein and Schwille, 2004). Further, single-molecule techniques usually need low concentration of labeled molecules and are normally applied to systems in equilibrium. One such single-molecule technique, which has been quite useful for membrane protein research is fluorescence correlation spectroscopy (FCS). It is a sensitive and powerful tool to measure important parameters like concentrations, mobility of molecules, equilibrium and rate constants for biomolecular interactions because FCS characterizes fluorescence intensity fluctuations in equilibrium (Haustein and Schwille, 2004; Enderlein et al., 2005; Ries and Schwille, 2012; Hink, 2015). FCS measurements are based on statistical correlation analyses of fluctuations in fluorescence intensity that arise from thousands of single-diffusion events of biomolecules through a femtolitre detection volume. FCS is therefore a suitable technique for monitoring the molecular binding events and aggregation.

Indeed, this technique has been used to study interfacial binding of peptides and proteins (Rusu et al., 2004; Rhoades et al., 2006) and the transmembrane insertion of proteins (Posokhov et al., 2008a,b; Melo et al., 2014) *in vitro* and *in vivo*. Further, FCS has been used to determine the dissociation constant for the F_1_b_2_ complex of *E. coli* ATP synthase (Diez et al., 2004a) and to study the dynamics of membrane receptors in live cells, which include CD8-induced ligand binding to T-cell receptor during T-cell activation (Gakamsky et al., 2005), determining the ligand binding constant to γ-aminobutyric acid A (GABA_A_) and to β_2_-adrenergic receptors (Meissner and Häberlein, 2003; Hegener et al., 2004). It has also been used in monitoring the conformational dynamics of H^+^-ATPase (Borsch et al., 1998). For *in vitro* studies of membrane proteins using FCS, giant unilamellar vesicles (GUVs) are an excellent model system because of their size similarity to eukaryotic cells and can therefore be visualized using a microscope (Méléard et al., 2009). Since efficient reconstitution of membrane proteins in GUVs is possible, FCS measurements in GUVs have been successfully carried out to monitor the light-induced mobility changes in bacteriorhodopsin (Kahya et al., 2002), lateral organization of mechanosensitive channel MscL, lactose transporter, ATP-binding cassette transport proteins etc. (Doeven et al., 2005). It should be noted that non-specific interactions between lipids and fluorescent probes can adversely affect the single molecule measurements of membrane proteins in liposomes and live cells. It is therefore important to choose the suitable fluorescent probe for single-molecule fluorescence studies in membranes and new insights have recently been provided to conduct experiments with minimal interference from the interactions between the probe and lipids in membranes (Zhang et al., 2017).

It is possible to use FCS to monitor the aggregation of labeled molecules, however, the sensitivity of autocorrelation analysis of aggregation/oligomerization process is restricted for molecules that form higher order clusters as shown in the case of 5HT3 receptor clustering (Pick et al., 2003). Monomer to dimer or tetramer transitions cannot be monitored by FCS because the diffusion coefficient of a spherical molecule depends on its hydrodynamic radius and thus on the cubic root of the molecular mass. The molecular mass between monomer and oligomer should therefore change by a factor of ~15–20 so as to get an appreciable difference in the diffusion coefficient of the molecule (Kahya, 2006). To overcome these limitations and to map specific biomolecular interactions, a variation of FCS namely the dual color FCS or fluorescence cross correlation spectroscopy (FCCS) is used, wherein the intensity fluctuations from two spectral channels that correspond to two labeled binding companions are cross-correlated (Haustein and Schwille, 2004; Bacia et al., 2006). The amplitude of the cross correlation function is highly sensitive to relative concentrations of the interacting partners diffusing together. The FCCS technique has been successfully employed to monitor dynamics of protein interactions (Larson et al., 2003) and to track the endocytic pathway of cholera toxin in live cells (Bacia et al., 2002). Combination of FCS with atomic force microscopy (AFM), which is a scanning probe high-resolution imaging technique, for the study of membranes and membrane-associated proteins has attracted a lot of attention (Chiantia et al., 2006a,b; Garcia-Saez and Schwille, 2007).

Two-photon excitation is advantageous for FCS studies (Berland et al., 1995; Schwille et al., 1999a,b). This is because the excitation volume is well defined and does not have usual problems like out-of-focus photobleaching and phototoxicity that are associated with single photon excitation. Further, the dynamic heterogeneities of the plasma membrane in cells have been probed using stimulated emission depletion (STED) microscopy (Eggeling et al., 2009). Importantly, combination of STED measurements using super-resolution microscopy can provide spatial resolution of ~50 nm, in other words, one can monitor the dynamics of membrane components at sub-diffraction resolution (Vicidomini et al., 2015; Sarkar and Chattopadhyay, 2019). In addition, FCS with total internal reflection fluorescence (TIRF) excitation has been used to study the immunoglobin binding kinetics to membranes (Thompson and Axelrod, 1983) and lateral mobility of membrane-binding proteins in live cell membranes (Ohsugi et al., 2006).

### Förster Resonance Energy Transfer

Förster resonance energy transfer or fluorescence resonance energy transfer (FRET) is the most widely used method to monitor inter- and intra-molecular interactions, oligomerization, physiologically-relevant conformational changes and elucidating dynamic protein interactions both *in vitro* and *in vivo* (Lakowicz, 2006; Roy et al., 2008; Taraska, 2012; Ma et al., 2014; Lerner et al., 2018). Further, FRET is also a powerful tool to monitor membranes (Loura and Prieto, 2011) and folding of membrane proteins (Kang et al., 2012), protein-lipid selectivity (Loura et al., 2010) and role of lipid-protein interactions in forming functional membrane protein oligomers (Schick et al., 2010; Gorbenko and Kinnunen, 2013). In general, there are two types of FRET namely the hetero-FRET (conventional) and homo-FRET. Determination of distances in the range of 10 to 100 Å between the donor and acceptor probes is possible using FRET as a “spectroscopic ruler” (Stryer, 1978). Since an average diameter of proteins (>50 KDa) is ~50–100 Å (Erickson, 2009), FRET is therefore a powerful tool for monitoring protein-protein interactions and their associated dynamic conformational transitions (Selvin, 1995; Geddes et al., 2006; Lerner et al., 2018).

#### Hetero-FRET

In conventional hetero-FRET, two probes (donor and acceptor) are used. FRET is a non-radiative energy transfer process between the excited state donor fluorophore and ground state acceptor molecule that quenches the fluorescence intensity of the donor. The term “resonance energy transfer (RET)” implies that the energy transfer is due to intermolecular dipole-dipole coupling, which means that this process does not involve emission and reabsorption of photons (Förster, 1965; Van Der Meer et al., 1994). The energy transfer depends on the spectral overlap between the donor and acceptor fluorescent molecules, the orientation of the transition moments of the probes, and most importantly, the distance between the two probes (Wu and Brand, 1994; Lakowicz, 2006). Basically, FRET is optimal when the distance range between two probes is ~0.6–1.3 R_o_, where R_o_ is the Förster distance (Selvin, 1995). The energy transfer can be detected either by the reduction of donor fluorescence intensity or lifetime or by an increase in acceptor fluorescence upon exciting at donor excitation wavelength. Observation of FRET clearly indicates some form of protein-protein interactions (oligomerization) in an ensemble cuvette-based measurement or on the cell surface (Sun et al., 2013; Khadria and Senes, 2015). A theoretical formalism has been developed by Veatch and Stryer (1977), which relates the size of the protein complex to the extent of FRET as a function of labeling ratio. This formalism has successfully been applied to confirm the dimeric nature of the gramicidin A transmembrane ion channel (Veatch and Stryer, 1977), the molecular oligomeric size of the colicin E1 channel in its closed state (Tory and Merrill, 1999), and bacterial multidrug ABC half-transporter BmrA (Dalmas et al., 2005). Among ion channels, the voltage-gated *Shaker* K^+^ channel has been the earliest system to be studied using FRET to monitor the voltage-driven movement of the voltage sensor (Cha et al., 1999; Glauner et al., 1999; Chanda et al., 2005). Since gating of ion channels usually involve complex structural dynamics changes, FRET has provided mechanistic insights into gating mechanisms of several ion channels, which include voltage-gated K_v_2.1 (Kobrinsky et al., 2006) and Ca_v_1.2 channels (Kobrinsky et al., 2003), L-type Ca^2+^ channels (Kobrinsky et al., 2004), chloride channels (Bykova et al., 2006; Ma et al., 2011), ryanodine receptors (George et al., 2006; Cornea et al., 2010), mechanosensitive channel MscL (Corry et al., 2005, 2010) etc. Recently, an interesting approach called FRET spectroscopy has been developed using spectral imaging (Raicu and Singh, 2013), where the pixel-by-pixel FRET efficiency is used to generate histograms. Information on distances and quaternary state can be obtained from this histogram since it relates to the statistical distribution of distances between fluorescent probes in oligomeric membrane proteins. This novel approach has already been applied to many membrane proteins, which include muscarinic M3 acetylcholine receptor (Patowary et al., 2013), ABC transporter (Singh et al., 2013), and sigma-1 receptor (Mishra et al., 2015). It should be noted that this approach can provide misleading information under certain conditions that exist simultaneously (Raicu and Singh, 2013).

#### Homo-FRET

In homo-FRET approach, a fluorescent probe with small Stokes shift is used. The same fluorescent probe acts as donor and acceptor in homo-FRET due to sufficient overlap between its absorption and emission (Lakowicz, 2006; Clayton, 2018). Unlike in conventional hetero-FRET, the intensity and/or the excited-state lifetime does not change in homo-FRET. Importantly, Weber (1960a,b) has discovered that homo-FRET results in depolarization, i.e., decrease of emission polarization, and homo-FRET efficiency is less effective when the fluorophore is excited at the red edge of its absorption spectrum. This “Weber red-edge effect” has been shown to be a general characteristic of homo-FRET (Weber and Shinitsky, 1970; Moens et al., 2004). However, as mentioned before, fluorescence polarization is strongly influenced by change in lifetimes as well as by protein motions (rotational diffusion), so care must be taken to ensure that the observed reduction in emission polarization is due to homo-FRET. Interestingly, a clever approach utilizing the “Weber red-edge effect” has been used to monitor homo-FRET between Trp in proteins (Moens et al., 2004). In this approach, the polarization values of Trp is measured using excitation at 295 and 310 nm (red-edge) and the 310/295 polarization ratio offers significant insights into homo-FRET between Trp residues. Further, in cellular studies, varying the extent of fluorophore labeling has been shown to be useful in detecting homo-FRET between proteins. Homo-FRET has been used to determine the clustering of GPI-anchored proteins (Varma and Mayor, 1998), epidermal growth factor receptor (Kozer et al., 2011) and serotonin 1A receptor (Ganguly et al., 2011) on the cell surface. Excellent reviews are available on FRET-based approaches to study the oligomerization of GPCRs (Gandia et al., 2008; Kaczor and Selent, 2011). Recently, the conformational plasticity in the KcsA K^+^ channel pore helix has been studied by homo-FRET of Trp fluorescence (Renart et al., 2019).

#### Variants of FRET

Several FRET approaches have been developed and each one has its own advantages and limitations (Sapsford et al., 2006; Ma et al., 2014; Kankanamge et al., 2019). They are luminescence resonance energy transfer (LRET), where lanthanide is used as a donor and a fluorophore is used as an acceptor. This has been used to monitor depolarization-induced voltage sensor movement (S4 helix) in *Shaker* K^+^ channels (Cha et al., 1999) and the topology of prokaryotic voltage-gated Na^+^ channel NaChBac and K^+^ channel KvAP (Richardson et al., 2006; Sandtner et al., 2007). Another FRET approach, bioluminescence resonance energy transfer (BRET), uses the light emitted (440–480 nm) from the reaction of luciferase enzyme with its substrate as donor light and a fluorescent label (usually a fluorescent protein) as the acceptor for energy transfer. Due to donor light obtained chemically in BRET instead of conventional optical excitation of donor, the problem of excitation crosstalk is therefore eliminated (Bacart et al., 2008; Gandia et al., 2008). BRET approach has been successfully used to characterize the structural rearrangements of the receptor associated with GPCR activation (Kankanamge et al., 2019). Another recent technique, transition metal ion FRET (tmFRET) employs a fluorophore as donor and a transition metal divalent cation (Ni^2+^, Co^2+^, or Cu^2+^) as non-fluorescent FRET acceptor to precisely measure interatomic distances (Taraska et al., 2009a,b; Yu et al., 2013). This method is suitable for measuring short distances of ~5–20 Å, and provides a sparse, dynamic measurement of structure using very low concentration of functional proteins in their native environment.

#### smFRET

Importantly, the strength of FRET approach is that it can be extended to the single molecule level (Weiss, 2000; Roy et al., 2008; Tan et al., 2014; Sasmal et al., 2016a; Castell et al., 2018; Lerner et al., 2018). Single-molecule FRET (smFRET) is a powerful tool to characterize distinct conformational states in macromolecules and the dynamics of their interconversion (Lerner et al., 2018). This involves monitoring the time-dependent structural changes in a single protein molecule that is labeled with a pair of donor and acceptor molecules at the site of interest (Weiss, 2000; Roy et al., 2008). Typically, the fluorophores used in smFRET studies have R_o_ of ~50 Å (Ma et al., 2017). The advantage of smFRET over ensemble FRET is that it can detect conformational dynamics and heterogeneity within a single protein molecule instead of measuring average interactions among protein molecules in an ensemble. Further, ensemble FRET measurements can average out the heterogeneous structural substates in a protein. This can be unmasked by smFRET approach, which can reveal valuable insights on structural dynamic changes in a protein (Roy et al., 2008). Since the first report of smFRET (Ha et al., 1996), its usage has exploded in recent times, particularly for membrane proteins (see Table 1). Some early smFRET studies of membrane proteins have studied the dimerization of gramicidin monomers (Borisenko et al., 2003) and proton-powered subunit rotation in a single membrane-bound F_o_F_1_-ATPase (Diez et al., 2004b). Further, smFRET has been used to monitor the conformational dynamics of the Na^+^-coupled aspartate transporter GltPh (Akyuz et al., 2013; Erkens et al., 2013), change in diameter during the opening and closing of the mechanosensitive ion channel MscL (Wang et al., 2014; Martinac, 2017). Recently, smFRET approach has been employed on the bacterial homolog of mammalian inwardly-rectifying potassium (KirBac) (Sadler et al., 2016; Wang et al., 2016). The gating behavior of the concatenated channels has been studied, which supports the “twist-to-shrink” mechanism for the channel closing. Further studies on KirBac1.1 reveal that the selectivity filter conformational dynamics is affected by ion occupancies (Wang et al., 2019). Similar studies have been carried out to study conformational dynamics of ligand-gated ion channels which include glutamate receptors (Vafabakhsh et al., 2015; MacLean et al., 2019) and N-methyl-D-aspartate (NMDA) receptor in living cells (Sasmal and Lu, 2014; Sasmal et al., 2016b). Further, smFRET measurements have also been used to monitor the structural dynamics of SNARE proteins involved in membrane fusion (Choi et al., 2010; Sakon and Weninger, 2010) and membrane transporters like LeuT (Zhao et al., 2010) and EmrE (Morrison et al., 2012) as they are involved in translocation of substrates across the membrane. For successful experiments using fluorescent proteins and FRET approaches, it is imperative to be familiar with their potential limitations, which are discussed in detail elsewhere (Piston and Kremers, 2007; Ma et al., 2014; Sasmal et al., 2016a).

**Table 1:** FRET studies of membrane proteins.

**Biological system**	**Donor**	**Acceptor**	**Förster radius (R_**o**_)**	**References**
Glycophorin A (GpA)	Pyrene	7-dimethyaminocoumarin	60 Å	Fisher et al., 1999
	Fluorescein	TAMRA	54 Å	Anbazhagan et al., 2010
Voltage-gated *Shaker* K^+^ channel	Fluorescein	TMR	55 Å	Glauner et al., 1999
Ryanodine receptor (RyR)	Alexa Fluor-488	Alexa Fluor-568	62 Å	Cornea et al., 2010
SLC4A1 anion exchanger	Alexa Fluor-488	TMR-MS	50 Å	Basu et al., 2010
Gramicidin	Cy3	Cy5	53 Å	Borisenko et al., 2003^*^
Leucine transporter (LeuT)	Cy3	Cy5	58.4 Å	Zhao et al., 2010^*^
Multidrug transporter (EmrE)	Alexa Fluor-488	Alexa Fluor-568	62 Å	Morrison et al., 2012^*^
Glutamate transporter	Alexa Fluor-488	TMR	50 Å	Koch and Larsson, 2005
	Cy3	Cy5	58.4 Å	Akyuz et al., 2013^*^
Neurotensin receptor 1 (NTS1)	ECFP	EYFP	45.6 Å	Harding et al., 2009
	mNeonGreen	ATTO-647	50 Å	Heitkamp et al., 2018^*^
Mechanosensitive channel MscL	Alexa Fluor-488	Alexa Fluor-568	62 Å	Corry et al., 2005, 2010; Wang et al., 2014^*^
NMDA receptor	Alexa Fluor-532	ATTO-594	50 Å	Sasmal and Lu, 2014^*^
Metabotropic glutamate receptors (mGluR)	DY-547	Alexa Fluor-647	52 Å	Vafabakhsh et al., 2015^*^
KirBac1.1	Alexa Fluor-555	Alexa Fluor-647	51 Å	Wang et al., 2016^*^
	Cy3B	Alexa Fluor-647	–	Sadler et al., 2016^*^
	Alexa Fluor-555	Alexa Fluor-647	51 Å	Wang et al., 2019^*^
β_2_ adrenergic receptor	Cy3B	Cy7	50.7 Å	Gregorio et al., 2017^*^
Cystic fibrosis transmembrane conductance regulator (CFTR)	ATTO-532	ATTO-647N	59 Å	Krainer et al., 2018^*^

*TMR, Tetramethylrhodamine; eCFP, Enhanced Cyan Fluorescent Protein; eYFP, Enhanced Yellow Fluorescent Protein; TAMRA, Tetramethylrhodamine Azide; TMR-MS, Tetramethylrhodamine methanesulfonate*.

* *smFRET studies*.

## Conclusion and Future Perspectives

Membrane proteins are essential for the viability of every cell and are important therapeutic targets (Bull and Doig, 2015). The dynamic nature of the lipid bilayer impairs the applicability of high-resolution structural studies using X-ray crystallography, NMR and electron microscopy. As a result, SDFL approaches have become an important tool for monitoring the organization and structural dynamics of membrane proteins under native conditions in real time, which is otherwise not obtained from crystallographic data. The power of SDFL approaches can be appreciated from the fact that the mechanisms of membrane protein function determined from fluorescence approaches have been effectively used to modify the structural model derived from cryoelectron microscopy (Johnson, 2005; Tilley et al., 2005). Despite being used for several decades, the fluorescence approaches continue to evolve to address the complex conformational and dynamic changes associated with the function of membrane proteins. For example, one-dimensional smFRET (measured only between two fluorophores) may not be sufficient to characterize the conformational dynamics of proteins that are intrinsically complex, highly heterogeneous and multidimensional. For this purpose, multidimensional smFRET has been developed to probe three sites of the macromolecule simultaneously (Hohng et al., 2004). Further, the recently developed surface-induced fluorescence attenuation (SIFA) has the potential to monitor both vertical and lateral dynamic motions of membrane proteins in supported membranes at the single molecule level (Ma et al., 2018). This means that monitoring the three dimensional movements of proteins is possible with these new SDFL approaches. In addition, the improvements in computational methods in utilizing the set of restraints from SDFL approaches play a key role in achieving atomistic-like structural models (Brunger et al., 2011; Kalinin et al., 2012). Interestingly, the structural information obtained from SDFL approaches are complementary to site-directed spin labeling and electron paramagnetic resonance (SDSL-EPR) approaches, particularly the Double Electron Electron Resonance (DEER) for distance measurements (Torbeev et al., 2009, 2011; Jeschke, 2012; White et al., 2013; Raghuraman et al., 2014). Since membrane proteins mediate majority of cellular functions and also play a crucial role in pathogenicity (Marinko et al., 2019), knowledge of structural and dynamic insights from SDFL approaches of membrane proteins will be pivotal to understand the cellular structure and function in physiological and pathological conditions and also for potential drug discovery.

## Author Contributions

SC and AD contributed to preparing figures. HR, SC, and AD wrote the manuscript.

### Conflict of Interest

The authors declare that the research was conducted in the absence of any commercial or financial relationships that could be construed as a potential conflict of interest.

## References

[B1] Akyuz N. Altman R. B. Blanchard S. C. Boudker O. (2013). Transport dynamics in a glutamate transporter homologue. Nature 502, 114–118. 10.1038/nature12265 23792560 PMC3829612

[B2] Alder N. N. Jensen R. E. Johnson A. E. (2008). Fluorescence mapping of mitochondrial TIM23 complex reveals a water-facing, substrate-interacting helix surface. Cell 134, 439–450. 10.1016/j.cell.2008.06.007 18692467

[B3] Alexiev U. Farrens D. L. (2014). Fluorescence spectroscopy of rhodopsins: insights and approaches. Biochim. Biophys. Acta 1837, 694–709. 10.1016/j.bbabio.2013.10.008 24183695 PMC3965647

[B4] Alexiev U. Rimke I. Pöhlmann T. (2003). Elucidation of the nature of the conformational changes of the EF-interhelical loop in bacteriorhodopsin and of the helix VIII on the cytoplasmic surface of bovine rhodopsin: a time-resolved fluorescence depolarization study. J. Mol. Biol. 328, 705–719. 10.1016/S0022-2836(03)00326-7 12706727

[B5] Anbazhagan V. Cymer F. Schneider D. (2010). Unfolding a transmembrane helix dimer: a FRET study in mixed micelles. Arch. Biochem. Biophys. 495, 159–164. 10.1016/j.abb.2010.01.006 20074546

[B6] Arora A. Raghuraman H. Chattopadhyay A. (2004). Influence of cholesterol and ergosterol on membrane dynamics: a fluorescence approach. Biochem. Biophys. Res. Commun. 318, 920–926. 10.1016/j.bbrc.2004.04.118 15147960

[B7] Bacart J. Corbel C. Jockers R. Bach S. Couturier C. (2008). The BRET technology and its application to screening assays. Biotechnol. J. 3, 311–324. 10.1002/biot.200700222 18228541

[B8] Bacia K. Kim S. A. Schwille P. (2006). Fluorescence cross-correlation spectroscopy in living cells. Nat. Methods 3, 83–89. 10.1038/nmeth822 16432516

[B9] Bacia K. Majoul I. V. Schwille P. (2002). Probing the endocytic pathway in live cells using dual-color fluorescence cross-correlation analysis. Biophys. J. 83, 1184–1193. 10.1016/S0006-3495(02)75242-9 12124298 PMC1302220

[B10] Bakheet T. M. Doig A. J. (2009). Properties and identification of human protein drug targets. Bioinformatics 25, 451–457. 10.1093/bioinformatics/btp002 19164304

[B11] Barrantes F. J. Antollini S. S. Blanton M. P. Prieto M. (2000). Topography of nicotinic acetylcholine receptor membrane-embedded domains. J. Biol. Chem. 275, 37333–37339. 10.1074/jbc.M005246200 10967108

[B12] Basu A. Mazor S. Casey J. R. (2010). Distance measurements with a concatamer of the plasma membrane Cl^−^/HCO^3−^ exchanger, AE1. Biochemistry 49, 9226–9240. 10.1021/bi101134h 20828148

[B13] Berezin M. Y. Achilefu S. (2010). Fluorescence lifetime measurements and biological imaging. Chem. Rev. 110, 2641–2684. 10.1021/cr900343z 20356094 PMC2924670

[B14] Berland K. M. So P. T. Gratton E. (1995). Two-photon fluorescence correlation spectroscopy: method and application to the intracellular environment. Biophys. J. 68, 694–701. 10.1016/S0006-3495(95)80230-4 7696520 PMC1281733

[B15] Blunck R. Cordero-Morales J. F. Cuello L. G. Perozo E. Bezanilla F. (2006). Detection of the opening of the bundle crossing in KcsA with fluorescence lifetime spectroscopy reveals the existence of two gates for ion conduction. J. Gen. Physiol. 128, 569–581. 10.1085/jgp.200609638 17043150 PMC2151582

[B16] Blunck R. McGuire H. Hyde H. C. Bezanilla F. (2008). Fluorescence detection of the movement of single KcsA subunits reveals cooperativity. Proc. Natl. Acad. Sci. U.S.A. 105, 20263–20268. 10.1073/pnas.0807056106 19074286 PMC2629256

[B17] Bohuszewicz O. Low H. H. (2018). Structure of a mitochondrial fission dynamin in the closed conformation. Nat. Struct. Mol. Biol. 25, 722–731. 10.1038/s41594-018-0097-6 30061604 PMC6104806

[B18] Borisenko V. Lougheed T. Hesse J. Fureder-Kitzmuller E. Fertig N. Behrends J. C.. (2003). Simultaneous optical and electrical recording of single gramicidin channels. Biophys. J. 84, 612–622. 10.1016/S0006-3495(03)74881-4 12524314 PMC1302642

[B19] Borsch M. Turina P. Eggeling C. Fries J. R. Seidel C. A. Labahn A. Graber P. (1998). Conformational changes of the H^+^-ATPase from Escherichia coli upon nucleotide binding detected by single molecule fluorescence. FEBS Lett. 437, 251–254. 10.1016/S0014-5793(98)01247-2 9824301

[B20] Brochon J. C. (1994). Maximum entropy method of data analysis in time-resolved spectroscopy. Methods Enzymol. 240, 262–311. 10.1016/S0076-6879(94)40052-0 7823835

[B21] Brunette A. M. J. Farrens D. L. (2014). Distance mapping in proteins using fluorescence spectroscopy: tyrosine, like tryptophan, quenches bimane fluorescence in a distance-dependent manner. Biochemistry 53, 6290–6301. 10.1021/bi500493r 25144569 PMC4196733

[B22] Brunger A. T. Strop P. Vrljic M. Chu S. Weninger K. R. (2011). Three-dimensional molecular modelling with single molecule FRET. J. Struct. Biol. 173, 497–505. 10.1016/j.jsb.2010.09.004 20837146 PMC3051805

[B23] Bull S. C. Doig A. J. (2015). Properties of protein drug target classes. PLoS ONE 10:e0117955. 10.1371/journal.pone.0117955 25822509 PMC4379170

[B24] Bykova E. A. Zhang X. D. Chen T. Y. Zheng J. (2006). Large movement in the C terminus of CLC-0 chloride channel during slow gating. Nat. Struct. Mol. Biol. 13, 1115–1119. 10.1038/nsmb1176 17115052

[B25] Callis P. R. (2014). Binding phenomena and fluorescence quenching. II: photophysics of aromatic residues and dependence of fluorescence spectra on protein conformation. J. Mol. Struct. 1077, 22–29. 10.1016/j.molstruc.2014.04.051

[B26] Callis P. R. Tusell J. R. (2014). MD + QM correlations with tryptophan fluorescence spectral shifts and lifetimes, in Fluorescence Spectroscopy and Microscopy: Methods and Protocols, Methods in Molecular Biology, eds Engelborghs Y. Visser A. J. W. G. (New York, NY: Springer Science+Business Media, LLC, 171–214. 10.1007/978-1-62703-649-8_8 24108627

[B27] Caputo G. A. London E. (2003). Using a novel dual fluorescence quenching assay for measurement of tryptophan depth within lipid bilayers to determine hydrophobic α-helix locations within membranes. Biochemistry 42, 3265–3274. 10.1021/bi026696l 12641458

[B28] Carpenter E. P. Beis K. Cameron A. D. Iwata S. (2008). Overcoming the challenges of membrane protein crystallography. Curr. Opin. Struct. Biol. 18, 581–586. 10.1016/j.sbi.2008.07.001 18674618 PMC2580798

[B29] Castell O. K. Dijkman P. M. Wiseman D. N. Goddard A. D. (2018). Single molecule fluorescence for membrane proteins. Methods 147, 221–228. 10.1016/j.ymeth.2018.05.024 29857189

[B30] Catici D. A. M. Amos H. E. Yang Y. van den Elsen J. M. H. Pudney C. R. (2016). The red edge excitation shift phenomenon can be used to unmask protein structural ensembles: implications for NEMO-ubiquitin interactions. FEBS J. 283, 2272–2284. 10.1111/febs.13724 27028374

[B31] Catterall W. A. (2010). Ion channel voltage sensors: structure, function, and pathophysiology. Neuron 67, 915–928. 10.1016/j.neuron.2010.08.021 20869590 PMC2950829

[B32] Cha A. Bezanilla F. (1997). Characterizing voltage-dependent conformational changes in the Shaker K^+^ channel with fluorescence. Neuron 19, 1127–1140. 10.1016/S0896-6273(00)80403-1 9390525

[B33] Cha A. Bezanilla F. (1998). Structural implications of fluorescence quenching in the Shaker K^+^ channel. J. Gen. Physiol. 112, 391–408. 10.1085/jgp.112.4.391 9758859 PMC2229426

[B34] Cha A. Snyder G. E. Selvin P. R. Bezanilla F. (1999). Atomic scale movement of the voltage-sensing region in a potassium channel measured via spectroscopy. Nature 402, 809–813. 10.1038/45552 10617201

[B35] Chanda B. Asamoah O. K. Bezanilla F. (2004). Coupling interactions between voltage sensors of the sodium channel as revealed by site-specific measurements. J. Mol. Biol. 123, 217–230. 10.1085/jgp.200308971 14981134 PMC2217449

[B36] Chanda B. Asamoah O. K. Blunck R. Roux B. Bezanilla F. (2005). Gating charge displacement in voltage-gated ion channels involves limited transmembrane movement. Nature 436, 852–856. 10.1038/nature03888 16094369

[B37] Chatterjee S. Das A. Raghuraman H. (2019). Biochemical and biophysical characterization of a prokaryotic Mg^2+^ ion channel: implications for cost-effective purification of membrane proteins. Protein Expr. Purif. 161, 8–16. 10.1016/j.pep.2019.04.005 31028884 PMC7116351

[B38] Chattopadhyay A. (1990). Chemistry and biology of N-(7-nitrobenz-2-oxa-1,3-diazol-4-yl)-labeled lipids: fluorescent probes of biological and model membranes. Chem. Phys. Lipids. 53, 1–15. 10.1016/0009-3084(90)90128-E 2191793

[B39] Chattopadhyay A. Haldar S. (2014). Dynamic insight into protein structure utilizing red edge excitation shift. Acc. Chem. Res. 47, 12–19. 10.1021/ar400006z 23981188

[B40] Chattopadhyay A. London E. (1987). Parallax method for direct measurement of membrane penetration depth utilizing fluorescence quenching by spin-labeled phospholipids. Biochemistry 26, 39–45. 10.1021/bi00375a006 3030403

[B41] Chattopadhyay A. London E. (1988). Spectroscopic and ionization properties of N-(7-nitrobenz-2-oxa-1,3-diazol-4-yl)-labeled lipids in model membranes. Biochim. Biophys. Acta 938, 24–34. 10.1016/0005-2736(88)90118-6 3337814

[B42] Chattopadhyay A. McNamee M. G. (1991). Average membrane penetration depth of tryptophan residues of the nicotinic acetylcholine receptor by the parallax method. Biochemistry 30, 7159–7164. 10.1021/bi00243a017 1854727

[B43] Chattopadhyay A. Mukherjee S. Raghuraman H. (2002). Reverse micellar organization and dynamics: a wavelength-selective fluorescence approach. J. Phys. Chem. B 106, 13002–13009. 10.1021/jp021801m

[B44] Chattopadhyay A. Raghuraman H. (2004). Application of fluorescence spectroscopy to membrane protein structure and dynamics. Curr. Sci. 87, 175–180.

[B45] Chen X. Wolfgang D. E. Sampson N. S. (2000). Use of the parallax-quench method to determine the position of the active-site loop of cholesterol oxidase in lipid bilayers. Biochemistry 39, 13383–13389. 10.1021/bi001407j 11063575

[B46] Chiantia S. Kahya N. Ries J. Schwille P. (2006a). Effects of ceramide on liquid-ordered domains investigated by simultaneous AFM and FCS. Biophys. J. 90, 4500–4508. 10.1529/biophysj.106.081026 16565041 PMC1471841

[B47] Chiantia S. Ries J. Kahya N. Schwille P. (2006b). Combined AFM and two-focus SFCS study of raft-exhibiting model membranes. Chem. Phys. Chem. 7, 2409–2418. 10.1002/cphc.200600464 17051578

[B48] Cho H. Stanzione F. Oak A. Kim G. H. Yerneni S. Qi L. (2019). Intrinsic structural features of the human IRE1a transmembrane domain sense membrane lipid saturation. Cell Rep. 27, 307–320. 10.1016/j.celrep.2019.03.017 30943411 PMC6467502

[B49] Choi U. B. Strop P. Vrljic M. Chu S. Brunger A. T. Weninger K. R. (2010). Single-molecule FRET-derived model of the synaptotagmin 1-SNARE fusion complex. Nat. Struct. Mol. Biol. 17, 318–324. 10.1038/nsmb.1763 20173763 PMC2922927

[B50] Chothia C. (1976). The nature of the accessible and buried surfaces in proteins. J. Mol. Biol. 105, 1–12. 10.1016/0022-2836(76)90191-1 994183

[B51] Christie M. P. Johnstone B. A. Tweten R. K. Parker M. W. Morton C. J. (2018). Choleterol-dependent cytolysins: from water-soluble state to membrane pore. Biophys. Rev. 10, 1337–1348. 10.1007/s12551-018-0448-x 30117093 PMC6233344

[B52] Chung L. A. Lear J. D. DeGrado W. F. (1992). Fluorescence studies of the secondary structure and orientation of a model ion channel peptide in phospholipid vesicles. Biochemistry 31, 6608–6616. 10.1021/bi00143a035 1378757

[B53] Clayton A. H. (2018). Fluorescence-based approaches for monitoring membrane receptor oligomerization. J. Biosci. 43, 463–469. 10.1007/s12038-018-9762-5 30002266

[B54] Cornea R. L. Nitu F. R. Montserrat S. Thomas D. D. Fruen B. R. (2010). Mapping the ryanodine receptor FK-506-binding protein subunit using fluorescence resonance energy transfer. J. Biol. Chem. 285, 19219–19226. 10.1074/jbc.M109.066944 20404344 PMC2885200

[B55] Cornette J. L. Cease K. B. Margalit H. Spouge J. L. Berzofsky J. A. DeLisi C. (1987). Hydrophobocity scales and computational techniques for detecting amphipathic structures in proteins. J. Mol. Biol. 195, 659–685. 10.1016/0022-2836(87)90189-6 3656427

[B56] Corry B. Hurst A. C. Pal P. Nomura T. Rigby P. Martinac B. (2010). An improved open-channel structure of MscL determined from FRET confocal microscopy and simulation. J. Gen. Physiol. 136, 483–494. 10.1085/jgp.200910376 20876362 PMC2947060

[B57] Corry B. Rigby P. Liu Z. W. Martinac B. (2005). Conformational changes involved in MscL channel gating measured using FRET spectroscopy. Biophys. J. 89, L49–L51. 10.1529/biophysj.105.072009 16199508 PMC1367003

[B58] Crowley K. Reinhart G. D. Johnson A. E. (1993). The signal sequence moves through a ribosomal tunnel into a noncytoplasmic aqueous environment at the ER membrane early in translocation. Cell 73, 1101–1115. 10.1016/0092-8674(93)90640-C 8513496

[B59] Crowley K. S. Liao S. Worrell V. E. Reinhart G. D. Johnson A. E. (1994). Secretory proteins move through the endoplasmic reticulum membrane via an aqueous, gated pore. Cell 78, 461–471. 10.1016/0092-8674(94)90424-3 8062388

[B60] Cuello L. G. Jogini V. Cortes D. M. Perozo E. (2010). Structural mechanism of C-type inactivation in K^+^ channels. Nature 466, 203–208. 10.1038/nature09153 20613835 PMC3033749

[B61] Daggett K. A. Sakmar T. P. (2011). Site-specific *in vitro* and *in vivo* incorporation of molecular probes to study G-protein-coupled receptors. Curr. Opin. Chem. Biol. 15, 392–398. 10.1016/j.cbpa.2011.03.010 21571577

[B62] Dalmas O. Do Cao M.-A. Lugo M. R. Sharom F. J. Di Pietro A. Jault M. A. (2005). Time-resolved fluorescence resonance energy transfer shows that the bacterial multidrug ABC half-transporter BmrA functions as a homodimer. Biochemistry 44, 4312–4321. 10.1021/bi0482809 15766260

[B63] Das A. Chatterjee S. Raghuraman H. (2019). Structural dynamics of the paddle motif loop in the activated conformation of KvAP voltage sensor. Biophys. J. 10.1016/j.bpj.2019.08.017. [Epub ahead of print]. PMC7036696 31547975

[B64] de Almeida R. F. Loura L. M. Prieto M. Watts A. Fedorov A. Barrantes F. J. (2004). Cholesterol modulates the organization of the γM4 transmembrane domain of the muscle nicotinic acetylcholine receptor. Biophys. J. 86, 2261–2272. 10.1016/S0006-3495(04)74284-8 15041665 PMC1304076

[B65] de Jesus A. J. Allen T. W. (2013). The determinants of hydrophobic mismatch for transmembrane helices. Biochim. Biophys. Acta 1828, 851–863. 10.1016/j.bbamem.2012.09.012 22995244

[B66] Deisenhofer J. Epp O. Miki K. Huber R. Michel H. (1985). Structure of the protein subunits in the photosynthetic reaction centre of *Rhodopseudomonas viridis* at 3Å resolution. Nature 318, 618–624. 10.1038/318618a0 22439175

[B67] Dekel N. Priest M. F. Parnas H. Parnas I. Bezanilla F. (2012). Depolarization induces a conformational change in the binding site region of the M2 muscarinic receptor. Proc. Natl. Acad. Sci. U.S.A. 109, 285–290. 10.1073/pnas.1119424109 22184214 PMC3252955

[B68] Demchenko A. P. (2002). The red-edge effects: 30 years of exploration. Luminescence 17, 19–42. 10.1002/bio.671 11816059

[B69] Demchenko A. P. (2008). Site-selective red-edge effects. Methods Enzymol. 450, 59–78. 10.1016/S0076-6879(08)03404-6 19152856

[B70] Demchenko A. P. Gallay J. Vincent M. Apell H. J. (1998). Fluorescence heterogeneity of tryptophans in Na,K-ATPase: evidences for temperature-dependent energy transfer. Biophys. Chem. 72, 265–283. 10.1016/S0301-4622(98)00107-0 9691270

[B71] Demchenko A. P. Mely Y. Duportail G. Klymchenko A. S. (2009). Monitoring biophysical properties of lipid membranes by environment-sensitive fluorescent probes. Biophys J. 96, 3461–3470. 10.1016/j.bpj.2009.02.012 19413953 PMC2711402

[B72] Demmers J. A. A. van Duijn E. Haverkamp J. Greathouse D. V. Koeppe R. E. I. I. Heck A. J. R.. (2001). Interfacial positioning and stability of transmembrane peptides in lipid bilayers studied by combining hydrogen/deuterium exchange and mass spectrometry. J Biol. Chem. 276, 34501–34508. 10.1074/jbc.M101401200 11435420

[B73] Diez M. Borsch M. Zimmermann B. Turina P. Dunn S. D. Graber P. (2004a). Binding of the *b*-subunit in the ATP synthase from *Escherichia coli*. Biochemistry 43, 1054–1064. 10.1021/bi0357098 14744151

[B74] Diez M. Zimmermann B. Borsch M. Konig M. Schweinberger E. Steigmiller S. (2004b). Proton-powered subunit rotation in single membrane-bound F_o_F_1_-ATP synthase. Nat. Struct. Mol. Biol. 11, 135–141. 10.1038/nsmb718 14730350

[B75] Doeven M. K. Folgering J. H. Krasnikov V. Geertsma E. R. van den B. G. Poolman B. (2005). Distribution, lateral mobility and function of membrane proteins incorporated into giant unilamellar vesicles. Biophys. J. 88, 1134–1142. 10.1529/biophysj.104.053413 15574707 PMC1305118

[B76] Doose S. Neuweiler H. Sauer M. (2009). Fluorescence quenching by photoinduced electron transfer: a reporter for conformational dynamics of macromolecules. Chem. Phys. Chem. 10, 1389–1398. 10.1002/cphc.200900238 19475638

[B77] Eftink M. R. (1991). Fluorescence quenching: theory and applications, in Topics in Fluorescence Spectroscopy, Vol. 2, ed Lakowicz J. R. (New York, NY: Plenum Press), 53–126. 10.1007/0-306-47058-6_2

[B78] Eftink M. R. Selvidge L. A. Callis P. R. Rehms A. A. (1990). Photophysics of indole derivatives: experimental resolution of L_a_ and L_b_ transitions and comparison with theory. J. Phys. Chem. 94, 3469–3479. 10.1021/j100372a022

[B79] Eggeling C. Ringemann C. Medda R. Schwarzmann G. Sandhoff K. Polyakova S.. (2009). Direct observation of the nanoscale dynamics of membrane lipids in a living cell. Nature 457, 1159–1162. 10.1038/nature07596 19098897

[B80] Eilers M. Patel A. B. Liu W. Smith S.O. (2002). Comparison of helix interactions in membrane and soluble α-bundle proteins. Biophys. J. 82, 2720–2736. 10.1016/S0006-3495(02)75613-0 11964258 PMC1302060

[B81] Elvington S. M. Bu F. Nichols J. W. (2005). Fluorescent, acyl chain-labeled phosphatidylcholine analogs reveal novel transport pathways across the plasma membrane of yeast. J. Biol. Chem. 280, 40957–40964. 10.1074/jbc.M507926200 16204231

[B82] Enderlein J. Gregor I. Patra D. Dertinger T. Kaupp U. B. (2005). Performance of fluorescence correlation spectroscopy for measuring diffusion and concentration. Chem. Phys. Chem 6, 2324–2336. 10.1002/cphc.200500414 16273566

[B83] Engelborghs Y. (2003). Correlating protein structure and protein fluorescence. J. Fluoresc. 13, 9–16. 10.1023/A:1022398329107

[B84] Erickson H. P. (2009). Size and shape of protein molecules at the nanometer level determined by sedimentation, gel filtration, and electron microscopy, in Biological Procedures Online, Vol. 11, ed Li S. (New York, NY: Springer), 32–51. 10.1007/s12575-009-9008-x PMC3055910 19495910

[B85] Erkens G. B. Hanelt I. Goudsmits J. M. Slotboom D. J. Oijen A. M. (2013). Unsynchronised subunit motion in single trimeric sodium-coupled aspartate transporters. Nature 502, 119–123. 10.1038/nature12538 24091978

[B86] Esposito R. Mensitieri G. de Nicola S. (2015). Improved maximum entropy method for the analysis of fluorescence spectroscopy data: evaluating zero-time shift and assessing its effect on the determination of fluorescence lifetimes. Analyst 140, 8138–8147. 10.1039/C5AN01811K 26541293

[B87] Fagerberg L. Jonasson K. von Heijne G. Uhlen M. Berglund L. (2010). Prediction of the human membrane proteome. Proteomics 10, 1141–1149. 10.1002/pmic.200900258 20175080

[B88] Feroz H. Kwon H. Peng J. Oh H. Ferlez B. Bakes C. S.. (2018). Improving the extraction and post-purification concentration of membrane proteins. Analyst 143, 1378–1386. 10.1039/C7AN01470H 29220051

[B89] Ferreon A. C. Gambin Y. Lemke E. A. Deniz A. A. (2009). Interplay of alpha-synuclein binding and conformational switching probed by single-molecule fluorescence. Proc. Natl. Acad. Sci. U.S.A. 106, 5645–5650. 10.1073/pnas.0809232106 19293380 PMC2667048

[B90] Fery-Forgues S. Fayet J.-P. Lopez A. J (1993). Drastic changes in the fluorescence properties of NBD probes with the polarity of the medium: involvement of a TICT state? J. Photochem. Photobiol. A 70, 229–243. 10.1016/1010-6030(93)85048-D

[B91] Fiserova E. Kubala M. (2012). Mean fluorescence lifetime and its error. J. Lumin. 132, 2059–2064. 10.1016/j.jlumin.2012.03.038

[B92] Fisher L. E. Engelman D. M. Sturgis J. N. (1999). Detergents modulate dimerization, but not helicity, of the Glycophorin A transmembrane domain. J. Mol. Biol. 293, 639–651. 10.1006/jmbi.1999.3126 10543956

[B93] Förster T. (1965). Delocalized excitation and excitation transfer, in Modern Quantum Chemistry, Vol. 3, ed Sinanoglu O. (New York, NY: Academic Press, 93–137.

[B94] Fu D. Libson A. Miercke L. J. Weitzman C. Nollert P. Krucinski J.. (2000). Structure of a glycerol-conducting channel and the basis for its selectivity. Science 290, 481–486. 10.1126/science.290.5491.481 11039922

[B95] Gakamsky D. M. Luescher I. F. Pramanik A. Kopito R. B. Lemonnier F. Vogel H.. (2005). CD8 kinetically promotes ligand binding to the T-cell antigen receptor. Biophys. J. 89, 2121–2133. 10.1529/biophysj.105.061671 15980174 PMC1366714

[B96] Gandia J. Luis C. Ferré S. Franco R. Ciruela F. (2008). Light resonance energy transfer-based methods in the study of G protein-coupled receptor oligomerization. Bioessays 30, 82–89. 10.1002/bies.20682 18081019

[B97] Ganguly S. Clayton A. H. Chattopadhyay A. (2011). Organization of higher-order oligomers of the serotonin1(A) receptor explored utilizing homo-FRET in live cells. Biophys. J. 100, 361–368. 10.1016/j.bpj.2010.12.3692 21244832 PMC3021661

[B98] Garcia-Saez A. J. Schwille P. (2007). Single molecule techniques for the study of membrane proteins. Appl. Microbiol. Biotechnol. 76, 257–266. 10.1007/s00253-007-1007-8 17497147

[B99] Geddes C. D. Lakowicz J. R. Clegg R. (2006). The history of FRET, in Reviews in Fluorescence, eds Geddes C. Lakowicz J. (New York, NY: Springer), 1–45. 10.1007/0-387-33016-X

[B100] George C. H. Jundi H. Walters N. Thomas N. L. West R. R. Lai F. A. (2006). Arrhythmogenic mutation-linked defects in ryanodine receptor autoregulation reveal a novel mechanism of Ca^2+^ release channel dysfunction. Circ. Res. 98, 88–97. 10.1161/01.RES.0000199296.70534.7c 16339485

[B101] Ghosh A. K. Rukmini R. Chattopadhyay A. (1997). Modulation of tryptophan environment in membrane-bound melittin by negatively charged phospholipids: implications in membrane organization and function. Biochemistry 36, 14291–14305. 10.1021/bi971933j 9398147

[B102] Giepmans B. N. G. Adams S. R. Ellisman M. H. Tsien R. Y. (2006). The fluorescent toolbox for assessing protein location and function. Science 312, 217–224. 10.1126/science.1124618 16614209

[B103] Glauner K. S. Mannuzzu L. M. Gandhi C. S. Isacoff E. Y. (1999). Spectroscopic mapping of voltage sensor movement in the Shaker potassium channel. Nature 402, 813–817. 10.1038/45561 10617202

[B104] Gorbenko G. Kinnunen P. K. J. (2013). FRET analysis of protein-lipid interactions, in Fluorescence Methods to Study Biological Membranes, Springer Series on Fluorescence, eds Mely Y. Duportail G. (Berlin; Heidelberg: Springer-Verlag, 115–140.

[B105] Gregorio G. G. Masureel M. Hilger D. Terry D. S. Juette M. Zhao H.. (2017). Single-molecule analysis of ligand efficacy in β_2_AR-G-protein activation. Nature 547, 68–73. 10.1038/nature22354 28607487 PMC5502743

[B106] Grigorieff N. Ceska T. A. Downing K. H. Baldwin J. M. Henderson R. (1996). Electron crystallographic refinement of the structure of bacteriorhodopsin. J. Mol. Biol. 259, 393–421. 10.1006/jmbi.1996.0328 8676377

[B107] Ha T. Enderle T. Ogletree D. F. Chemla D. S. Selvin P. R. Weiss S. (1996). Probing the interaction between two single molecules: fluorescence resonance energy transfer between a single donor and a single acceptor. Proc. Natl. Acad. Sci. U.S.A. 93, 6264–6268. 10.1073/pnas.93.13.6264 8692803 PMC39010

[B108] Haldar S. Kombrabail M. Krishnamoorthy G. Chattopadhyay A. (2010a). Monitoring membrane protein conformational heterogeneity by fluorescence lifetime distribution analysis using the maximum entropy method. J. Fluoresc. 20, 407–413. 10.1007/s10895-009-0554-z 19816758

[B109] Haldar S. Raghuraman H. Chattopadhyay A. (2008). Monitoring orientation and dynamics of membrane-bound melittin utilizing dansyl fluorescence. J. Phys. Chem. B 112, 14075–14082. 10.1021/jp805299g 18842019

[B110] Haldar S. Raghuraman H. Namani T. Rajarathnam Chattopadhyay A. (2010b). Membrane interaction of the N-terminal domain of chemokine receptor CXCR1. Biochim Biophys. Acta 1798, 1056–1061. 10.1016/j.bbamem.2010.02.029 20226759

[B111] Harding P. J. Attrill H. Boehringer J. Rose S. Wadhams G. H. Smith E.. (2009). Constitutive dimerization of the G-protein coupled receptor, neurotensin receptor 1, reconstituted into phospholipid bilayers. Biophys. J. 96, 964–973. 10.1016/j.bpj.2008.09.054 19186134 PMC2716571

[B112] Hattori M. Iwase N. Furuya N. Tanaka Y. Tsukazaki T. Ishitani R. (2009). Mg^2+^-dependent gating of bacterial MgtE channel underlies Mg^2+^ homeostasis. EMBO J. 28, 3602–3612. 10.1038/emboj.2009.288 19798051 PMC2782099

[B113] Haugland R. P. (2005). The Handbook – A Guide to Fluorescent Probes and Labeling Technologies (Molecular Probes), 10th Edn. Eugene, OR: Molecular Probes Inc.

[B114] Haustein E. Schwille P. (2004). Single-molecule spectroscopic methods. Curr. Opin. Struct. Biol. 14, 531–540. 10.1016/j.sbi.2004.09.004 15465312

[B115] Hawe A. Sutter M. Jiskoot W. (2008). Extrinsic fluorescent dyes as tools for protein characterization. Pharm. Res. 25, 1487–1499. 10.1007/s11095-007-9516-9 18172579 PMC2440933

[B116] Hegener O. Prenner L. Runkel F. Baader S. L. Kappler J. Häberlein H. (2004). Dynamics of β_2_-adrenergic receptor-ligand complexes on living cells. Biochemistry 43, 6190–6199. 10.1021/bi035928t 15147203

[B117] Heitkamp T. Grisshammer R. Börsch M. (2018). Towards monitoring conformational changes of the GPCR neurotensin receptor 1 by single-molecule FRET. Proc. SPIE Int. Soc. Opt. Eng. 10498:104980T. 30013286 10.1117/12.2286787 PMC6044442

[B118] Hendrickson W. A. (2016). Atomic-level analysis of membrane-protein structure. Nat. Struct. Mol. Biol. 23, 464–467. 10.1038/nsmb.3215 27273628 PMC5299386

[B119] Henzler-Wildman K. Kern D. (2007). Dynamic personalities of proteins. Nature 450, 964–972. 10.1038/nature06522 18075575

[B120] Heuck A. P. Hotze E. M. Tweten R. K. Johnson A. E. (2000). Mechanism of membrane insertion of a multimeric β-barrel protein: perfringolysin O creates a pore using ordered and coupled conformational changes. Mol. Cell 6, 1233–1242. 10.1016/S1097-2765(00)00119-2 11106760

[B121] Heuck A. P. Johnson A. E. (2002). Pore-forming protein structure analysis in membranes using multiple independent fluorescence techniques. Cell Biochem. Biophys. 36, 89–101. 10.1385/CBB:36:1:89 11939373

[B122] Hink M. A. (2015). Fluorescence correlation spectroscopy, in Advanced Fluorescence Microscopy. Methods in Molecular Biology, ed Verveer P. J. (New York, NY: Springer, 135–150. 10.1007/978-1-4939-2080-8_8 25391798

[B123] Ho D. Lugo M. R. Merrill A. R. (2013). Harmonic analysis of the fluorescence response of bimane adducts of colicin E1 at helices 6, 7, and 10. J. Biol. Chem. 288, 5136–5148. 10.1074/jbc.M112.436303 23264635 PMC3576118

[B124] Hohng S. Joo C. Ha T. (2004). Single-molecule three-color FRET. Biophys. J. 87, 1328–1337. 10.1529/biophysj.104.043935 15298935 PMC1304471

[B125] Homan R. Eisenberg M. (1985). A fluorescence quenching technique for the measurement of paramagnetic ion concentrations at the membrane/water interface. Intrinsic and X537A-mediated cobalt fluxes across lipid bilayer membranes. Biochim. Biophys. Acta 812, 485–492. 10.1016/0005-2736(85)90323-2 2981549

[B126] Islas L. D. Zagotta W. N. (2006). Short-range molecular rearrangements in ion channels detected by tryptophan quenching of bimane fluorescence. J. Gen. Physiol. 128, 337–346. 10.1085/jgp.200609556 16940556 PMC2151569

[B127] James N. G. Jameson D. M. (2014). Steady-state fluorescence polarization/anisotropy for the study of protein interactions, in Fluorescence Spectroscopy and Microscopy: Methods and Protocols, Methods in Molecular Biology, eds Engelborghs Y. Visser A. J. W. G. (New York, NY: Spinger Science+Business Media, LLC, 29–42. 10.1007/978-1-62703-649-8_2 24108621

[B128] Jameson D. M. Ross J. A. (2010). Fluorescence polarization/anisotropy in diagnostics and imaging. Chem. Rev. 110, 2685–2708. 10.1021/cr900267p 20232898 PMC2868933

[B129] Janz J. M. Farrens D. L. (2004). Rhodopsin activation exposes a key hydrophobic binding site for the transducin α-subunit C terminus. J. Biol. Chem. 279, 29767–29773. 10.1074/jbc.M402567200 15070895

[B130] Jeschke G. (2012). DEER distance measurements on proteins. Annu. Rev. Phys. Chem. 63, 419–446. 10.1146/annurev-physchem-032511-143716 22404592

[B131] Jha S. K. Dhar D. Krishnamoorthy G. Udgaonkar J. B. (2009). Continuous dissolution of structure during the unfolding of a small protein. Proc. Natl. Acad. Sci. U.S.A. 106, 11113–11118. 10.1073/pnas.0812564106 19553216 PMC2708691

[B132] Jiang Y. Lee A. Chen J. Ruta V. Cadene M. Chait B. T. MacKinnon R. (2003). X-ray structure of a voltage-dependent K^+^ channel. Nature 423, 33–41. 10.1038/nature01580 12721618

[B133] Jittikoon J. East M. J. Lee A. G. (2007). A fluorescence method to define transmembrane α-helices in membrane proteins: studies with bacterial diacylglycerol kinase. Biochemistry 46, 10950–10959. 10.1021/bi7008213 17722884

[B134] Johnson A. E. (2005). Fluorescence approaches for determining protein conformations, interactions and mechanisms at membranes. Traffic 6, 1078–1092. 10.1111/j.1600-0854.2005.00340.x 16262720

[B135] Johnson C. K. Osborn K. D. Allen M. W. Slaughter B. D. (2005). Single-molecule fluorescence spectroscopy: new probes of protein function and dynamics. Physiology 20, 10–14. 10.1152/physiol.00037.2004 15653834

[B136] Kaback H. R. Wu J. (1999). What to do while awaiting crystals of a membrane transport protein and thereafter. Acc. Chem. Res. 32, 805–813. 10.1021/ar970256i

[B137] Kaczor A. A. Selent J. (2011). Oligomerization of G-protein-coupled receptors: biochemical and biophysical methods. Curr. Med. Chem. 18, 4606–4634. 10.2174/092986711797379285 21864280

[B138] Kahya N. (2006). Targeting membrane proteins to liquid-ordered phases: molecular self-organization explored by fluorescence correlation spectroscopy. Chem. Phys. Lipids 141, 158–168. 10.1016/j.chemphyslip.2006.02.026 16696961

[B139] Kahya N. Wiersma D. A. Poolman B. Hoekstra D. (2002). Spatial organization of bacteriorhodopsin in model membranes. Light-induced mobility changes. J. Biol. Sci. 277, 39304–39311. 10.1074/jbc.M202635200 12167614

[B140] Kale J. Chi X. Leber B. Andrews D. (2014). Examining the molecular mechanism of Bcl-2 family proteins at membranes by fluorescence spectroscopy. Meth. Enzymol. 544, 1–23. 10.1016/B978-0-12-417158-9.00001-7 24974284

[B141] Kalinin S. Peulen T. Sindbert S. Rothwell P. J. Berger S. Restle T.. (2012). A toolkit and benchmark study for FRET-restrained high-precision structural modeling. Nat. Methods 9, 1218–1225. 10.1038/nmeth.2222 23142871

[B142] Kang G. Lopez-Pena I. Oklejas V. Gary C. S. Cao W. Kim J. E. (2012). Förster resonance energy transfer as a probe of membrane protein folding. Biochim. Biophys. Acta 1818, 154–161. 10.1016/j.bbamem.2011.08.029 21925139 PMC3253952

[B143] Kankanamge D. Ratnayake K. Senarath K. Tennakoon M. Harmon E. Karunarathne A. (2019). Optical approaches for single-cell and subcellular analysis of GPCR-G protein signaling. Anal. Bioanal. Chem. 411, 4481–4508. 10.1007/s00216-019-01774-6 30927013 PMC6612303

[B144] Khadria A. S. Senes A. (2015). Fluorophores, environments, and quantification techniques in the analysis of transmembrane helix interaction using FRET. Biopolymers 104, 247–264. 10.1002/bip.22667 25968159 PMC4516689

[B145] Killian J. A. von Heijne G. (2000). How proteins adapt to a membrane-water interface. Trends Biochem. Sci. 25, 429–434. 10.1016/S0968-0004(00)01626-1 10973056

[B146] Kim A. Cross T. A. (2002). Uniformity, ideality, and hydrogen bonds in transmembrane α-helices. Biophys. J. 83, 2084–2095. 10.1016/S0006-3495(02)73969-6 12324426 PMC1302297

[B147] Kim Y. Ho S. O. Gassman N. R. Korlann Y. Landorf E. V. Collart F. R. Weiss S. (2008). Efficient site-specific labeling of proteins via cysteines. Bioconjugate Chem. 19, 786–791. 10.1021/bc7002499 18275130 PMC3086356

[B148] Kobayashi T. Pagano R. E. (1988). ATP-dependent fusion of liposomes with the Golgi apparatus of perforated cells. Cell 55, 797–805. 10.1016/0092-8674(88)90135-3 3191530

[B149] Kobrinsky E. Kepplinger K. J. Yu A. Harry J. B. Kahr H. Romanin C. (2004). Voltage-gated rearrangements associated with differential β-subunit modulation of the L-type Ca^2+^ channel inactivation. Biophys. J. 87, 844–857. 10.1529/biophysj.104.041152 15298893 PMC1304494

[B150] Kobrinsky E. Schwartz E. Abernethy D. R. Soldatov N. M. (2003). Voltage-gated mobility of the Ca^2+^ channel cytoplasmic tails and its regulatory role. J. Biol. Chem. 278, 5021–5028. 10.1074/jbc.M211254200 12473653

[B151] Kobrinsky E. Stevens L. Kazmi Y. Wray D. Soldatov N. M. (2006). Molecular rearrangements of the K_v_2.1 potassium channel termini associated with voltage gating. J. Biol. Chem. 281, 19233–19240. 10.1074/jbc.M601231200 16690619

[B152] Koch H. P. Larsson H. P. (2005). Small-scale molecular motions accomplish glutamate uptake in human glutamate transporters. J. Neurosci. 25, 1730–1736. 10.1523/JNEUROSCI.4138-04.2005 15716409 PMC6725926

[B153] Koehorst R. B. M. Spruijt R. B. Hemminga M. A. (2008). Site-directed fluorescence labeling of a membrane protein with BADAN: probing protein topology and local environment. Biophys. J. 94, 3945–3955. 10.1529/biophysj.107.125807 18234831 PMC2367197

[B154] Kohout S. C. Bell S. C. Liu L. Xu Q. Minor D. L. Jr. Isacoff E.Y. (2010). Electrochemical coupling in the voltage-dependent phosphatase Ci-VSP. Nat. Chem. Biol. 6, 369–375. 10.1038/nchembio.349 20364128 PMC2857593

[B155] Kosower E. M. Kanety H. Dodluk H. Hermolin J. (1982). Bimanes. 9. Solvent and substitution effects on intramolecular charge-transfer quenching of the fluorescence of *syn*-1,5-diazabicyclo[3.3.0]octadienediones (*syn*-9,10-dioxabimanes). J. Phys. Chem. 86, 1270–1277. 10.1021/j100397a013

[B156] Kozer N. Kelly M. P. Orchard S. Burgess A. W. Scott A. W. Clayton A. H. (2011). Differential and synergistic effects of epidermal growth factor receptor antibodies on unliganded ErbB dimers and oligomers. Biochemistry 50, 3581–3590. 10.1021/bi101785h 21495621

[B157] Krainer G. Treff A. Hartmann A. Stone T. A. Schenkel M. Keller S.. (2018). A minimal helical-hairpin motif provides molecular insights into misfolding and pharmacological rescue of CFTR. Commun. Biol. 1:154. 10.1038/s42003-018-0153-0 30302398 PMC6162264

[B158] Kratochvil H. T. Carr J. K. Matulef K. Anne A. W. Li H. Maj M.. (2016). Instantaneous ion configurations in the K^+^ ion channel selectivity filter revealed by 2D IR spectroscopy. Science 353, 1040–1044. 10.1126/science.aag1447 27701114 PMC5544905

[B159] Krepkiy D. Mihailescu M. Freitas J. A. Schow E. V. Worcester D. L. Gawrisch K.. (2009). Structure and hydration of membranes embedded with voltage-sensing domains. Nature 462, 473–479. 10.1038/nature08542 19940918 PMC2784928

[B160] Krishnamoorthy G. (2018a). Fluorescence lifetime distribution brings out mechanisms involving biomolecules while quantifying population heterogeneity, in Reviews in Fluorescence 2017, ed Geddes C. D. (New York, NY: Springer, 75–98.

[B161] Krishnamoorthy G. (2018b). Fluorescence spectroscopy for revealing mechanisms in biology: strengths and pitfalls. J. Biosci. 43, 555–567. 10.1007/s12038-018-9763-4 30002272

[B162] Kuo A. Gulbis J. M. Antcliff J. F. Rahman T. Lowe E. D. Zimmer J.. (2003). Crystal structure of the potassium channel KirBac1.1. in the closed state. Science 300, 1922–1926. 10.2210/pdb1p7b/pdb 12738871

[B163] Kyrychenko A. Lim N. M. Vasquez-Montes V. Rodnin M. V. Freites J. A. Nguyen L. P.. (2018). Refining protein penetration into the lipid bilayer using fluorescence quenching and molecular dynamics simulations: the case of diphtheria toxin translocation domain. J. Membr. Biol. 251, 379–391. 10.1007/s00232-018-0030-2 29550876 PMC6030514

[B164] Kyrychenko A. V. Ladokhin A. S. (2018). Fluorescence tools for studies of membrane protein insertion. Biopolym. Cell 34, 251–271. 10.7124/bc.00097F

[B165] Ladokhin A. S. (1997). Distribution analysis of depth-dependent fluorescence quenching in membranes: a practical guide. Methods Enzymol. 278, 462–473. 10.1016/S0076-6879(97)78024-8 9170327

[B166] Ladokhin A. S. (1999). Evaluation of lipid exposure of tryptophan residues in membrane peptides and proteins. Anal. Biochem. 276, 65–71. 10.1006/abio.1999.4343 10585745

[B167] Ladokhin A. S. (2014). Measuring membrane penetration with depth-dependent fluorescence quenching: distribution analysis is coming of age. Biochim. Biophys. Acta 1838, 2289–2295. 10.1016/j.bbamem.2014.02.019 24593994 PMC4082754

[B168] Ladokhin A. S. Jayasinghe S. White S. H. (2000). How to measure and analyse tryptophan fluorescence in membranes properly, and why bother? Anal. Biochem. 285, 235–245. 10.1006/abio.2000.4773 11017708

[B169] Ladokhin A. S. Wang L. Steggles A. W. Holloway P. W. (1991). Fluorescence study of a mutant cytochrome b5 with a single tryptophan in the membrane-binding domain. Biochemistry 30, 10200–10206. 10.1021/bi00106a018 1931948

[B170] Ladokhin A. S. White S. H. (1999). Folding of amphipathic alpha-helices on membranes: energetics of helix formation by melittin. J. Mol. Biol. 285, 1363–1369. 10.1006/jmbi.1998.2346 9917380

[B171] Lakowicz J. R. (2006). Principles of Fluorescence Spectroscopy, 3rd Edn. New York, NY: Springer.

[B172] Lakshmikanth G. S. Sridevi K. Krishnamoorthy G. Udgaonkar J. B. (2001). Structure is lost incrementally during the unfolding of barstar. Nat. Struct. Biol. 8, 799–804. 10.1038/nsb0901-799 11524685

[B173] Larson D. R. Ma Y. M. Vogt V. M. Webb W. W. (2003). Direct measurement of Gag-Gag interactions during retrovirus assembly with FRET and fluorescence correlation spectroscopy. J. Cell Biol. 162, 1233–1244. 10.1083/jcb.200303200 14517204 PMC2173966

[B174] Larsson H. P. Tzingounis A. V. Koch H. P. Kavanaugh M. P. (2004). Fluorometric measurements of conformational changes in glutamate transporters. Proc. Natl. Acad. Sci. U.S.A. 101, 3951–3956. 10.1073/pnas.0306737101 15001707 PMC374350

[B175] Lehrer S. S. (1971). Solute perturbation of protein fluorescence. The quenching of the tryptophyl fluorescence of model compounds and of lysozyme by iodide ion. Biochemistry 10, 3254–3263. 10.1021/bi00793a015 5119250

[B176] Lerner E. Cordes T. Ingargiola A. Alhadid Y. Chung S. Y. Michalet X. Weiss S. (2018). Toward dynamic structural biology: two decades of single-molecule Förster resonance energy transfer. Science 359:eaan1133. 10.1126/science.aan1133 29348210 PMC6200918

[B177] Li T. Hassanali A. A. Kao Y. T. Zhong D. Singer S. J. (2007). Hydration dynamics and time scales of coupled water-protein fluctuations. J. Am. Chem. Soc. 129, 3376–3382. 10.1021/ja0685957 17319669

[B178] Liao S. Lin J. Do H. Johnson A. E. (1997). Both lumenal and cytosolic gating of the aqueous ER translocon pore are regulated from inside the ribosome during membrane protein integration. Cell 90, 31–41. 10.1016/S0092-8674(00)80311-6 9230300

[B179] Lin P.-J. Jongsma C. G. Liao S. Johnson A. E. (2011). Transmembrane segments of nascent polytopic membrane proteins control cytosolic/ER targeting during membrane integration. J. Cell Biol. 195, 41–54. 10.1083/jcb.201103117 21949411 PMC3187712

[B180] Lin S. Struve W. S. (1991). Time-resolved fluorescence of nitrobenzoxadiazol-aminohexanoic acid: effect of intermolecular hydrogen bonding on non-radiative decay *Photochem*. Photobiol. 54, 361–365. 10.1111/j.1751-1097.1991.tb02028.x 1784635

[B181] Liu R. Sharom F. J. (1996). Site-directed fluorescence labeling of P-glycoprotein on cysteine residues in the nucleotide binding domains. Biochemistry 35, 11865–11873. 10.1021/bi960823u 8794769

[B182] London E. Ladokhin A. S. (2002). Measuring the depth of amino acid residues in membrane-inserted peptides by fluorescence quenching, in Current Topics in Membranes, eds Benos D. Simons S. (San Diego, CA: Elsevier, 89–115.

[B183] Loura L. M. S. Prieto M. (2011). FRET in membrane biophysics: an overview. Front. Physiol. 2:82. 10.3389/fphys.2011.00082 22110442 PMC3216123

[B184] Loura L. M. S. Prieto M. Fernandes F. (2010). Quantification of protein-lipid selectivity using FRET. Eur. Biophys. J. 39, 565–578. 10.1007/s00249-009-0532-z 20238256 PMC2841278

[B185] Ma J. Yanez-Orozco I. S. Adariani S. R. Dolino D. Jayaraman V. Sanabria H. (2017). High precision FRET at single-molecule level for biomolecule structure determination. J. Vis. Exp. 123, 55623 10.3791/55623 PMC5607938 28570518

[B186] Ma L. Li Y. Ma J. Hu S. Li M. (2018). Watching three-dimensional movements of single membrane proteins in lipid bilayers. Biochemistry 57, 4735–4740. 10.1021/acs.biochem.8b00253 29619828

[B187] Ma L. Rychkov G. Y. Bykova E. A. Zheng J. Bretag A. H. (2011). Movement of hClC-1 C-termini during common gating and limits on their cytoplasmic location. Biochem. J. 436, 415–428. 10.1042/BJ20102153 21413926

[B188] Ma L. Yang F. Zheng J. (2014). Application of fluorescence resonance energy transfer in protein studies. J. Mol. Struct. 1077, 87–100. 10.1016/j.molstruc.2013.12.071 25368432 PMC4215735

[B189] MacLean D. M. Durham R. J. Jayaraman V. (2019). Mapping the conformational landscape of glutamate receptors using single molecule FRET. Trends Neurosci. 42, 128–139. 10.1016/j.tins.2018.10.003 30385052 PMC6359962

[B190] Malovrh P. Viero G. Serra M. D. Podlesek Z. Lakey J. H. Macek P.. (2003). A novel mechanism of pore formation: membrane penetration by the N-terminal amphipathic region of equinatoxin. J. Biol. Chem. 278, 22678–22685. 10.1074/jbc.M300622200 12676945

[B191] Mannuzzu L. M. Moronne M. M. Isacoff E. Y. (1996). Direct physical measure of conformational rearrangement underlying potassium channel gating. Science 271, 213–216. 10.1126/science.271.5246.213 8539623

[B192] Mansoor S. E. DeWitt M. A. Farrens D. L. (2010). Distance mapping in proteins using fluorescence spectroscopy: the tryptophan-induced quenching (TrIQ) method. Biochemistry 49, 9722–9731. 10.1021/bi100907m 20886836 PMC3938424

[B193] Mansoor S. E. Farrens D. L. (2004). High-throuoghput protein structural analysis using site-directed fluorescence labeling and the bimane derivative (2-pyridyl)dithiobimane. Biochemistry 43, 9426–9438. 10.1021/bi036259m 15260485

[B194] Mansoor S. E. Mchaourab H. S. Farrens D. L. (2002). Mapping proximity within proteins using fluorescence spectroscopy. A study of T4 lysozyme showing that the tryptophan residues quench bimane fluorescence. Biochemistry 41, 2475–2484. 10.1021/bi011198i 11851393

[B195] Marinko J. Huang H. Penn W. D. Capra J. A. Schlebach J. P. Sanders C. R. (2019). Folding and misfolding of human membrane proteins in health and disease: from single molecules to cellular proteostasis. Chem. Rev. 119, 5537–5606. 10.1021/acs.chemrev.8b00532 30608666 PMC6506414

[B196] Martinac B. (2017). Single-molecule FRET studies of ion channels. Prog. Biophys. Mol. Biol. 130, 192–197. 10.1016/j.pbiomolbio.2017.06.014 28648629

[B197] Meers P. (1990). Location of tryptophans in membrane-bound annexins. Biochemistry 29, 3325–3330. 10.1021/bi00465a025 2139793

[B198] Meissner O. Häberlein H. (2003). Lateral mobility and specific binding to GABA_A_ receptors on hippocampal neurons monitored by fluorescence correlation spectroscopy. Biochemistry 42, 1667–1672. 10.1021/bi0263356 12578381

[B199] Méléard P. Bagatolli L. A. Pott T. (2009). Giant unilamellar vesicle electroformation from lipid mixtures to native membranes under physiological conditions. Methods Enzymol. 465, 161–176. 10.1016/S0076-6879(09)65009-6 19913167

[B200] Melo A. M. Prieto M. Coutinho A. (2014). Quantifying lipid- protein interaction by fluorescence correlation spectroscopy (FCS), in Fluorescence Spectroscopy and Microscopy. Methods in Molecular Biology (Methods and Protocols), eds Engelborghs Y. Visser A. J. W. G. (New York, NY: Humana Press),575–595. 10.1007/978-1-62703-649-8_26 24108645

[B201] Mishra A. K. Mavlyutov T. Singh D. R. Biener G. Yang J. Oliver J. A.. (2015). The sigma-1 receptors are present in monomeric and oligomeric forms in living cells in the presence and absence of ligands. Biochem. J. 466, 263–271. 10.1042/BJ20141321 25510962 PMC4500508

[B202] Mishra P. Jha S. K. (2019). Slow motion protein dance visualized using red-edge excitation shift of a buried fluorophore. J. Phys. Chem. B. 123, 1256–1264. 10.1021/acs.jpcb.8b11151 30640479

[B203] Moens P. D. J. Helms M. K. Jameson D. M. (2004). Detection of tryptophan energy transfer in proteins. Protein J. 23, 79–83. 10.1023/B:JOPC.0000016261.97474.2e 15115185

[B204] Moraes I. Evans G. Sanchez-Weatherby J. Newstead S. Stewart P. D. S. (2014). Membrane protein structure determination-The next generation. Biochim. Biophys. Acta 1838, 78–87. 10.1016/j.bbamem.2013.07.010 23860256 PMC3898769

[B205] Morris S. J. Bradley D. Blumenthal R. (1985). The use of cobalt ions as collisional quencher to probe surface charge and stability of fluorescently labelled bilayer vesicles. Biochim. Biophys. Acta 818, 365–372. 10.1016/0005-2736(85)90011-2 4041444

[B206] Morrison E. A. DeKoster G. T. Dutta S. Clarkson M. W. Vafabakhsh R. Bahl A.. (2012). Antiparallel EmrE exports drugs by exchanging between asymmetric structures. Nature 481, 45–50. 10.1038/nature10703 22178925 PMC3253143

[B207] Mukherjee S. Chattopadhyay A. (2005). Monitoring cholesterol organization in membranes at low concentrations utilizing the wavelength-selective fluorescence approach. Chem. Phys. Lipids 134, 79–84. 10.1016/j.chemphyslip.2004.12.001 15752466

[B208] Mukherjee S. Chattopadhyay A. Samanta A. Soujanya T. (1994). Dipole moment change of NBD group upon excitation studied using solvatochromic and quantum chemical approaches: Implications in membrane research. J. Phys. Chem. 98, 2809–2812. 10.1021/j100062a014

[B209] Mukherjee S. Kalipatnapu S. Pucadyil T. J. Chattopadhyay A. (2006). Monitoring the organization and dynamics of bovine hippocampal membranes utilizing differentially localized fluorescent membrane probes. Mol. Memb. Biol. 23, 430–441. 10.1080/09687860600803223 17060160

[B210] Mukherjee S. Kombrabail M. Krishnamoorthy G. Chattopadhyay A. (2007a). Dynamics and heterogeneity of bovine hippocampal membranes: role of cholesterol and proteins. Biochim. Biophys. Acta 1768, 2130–2144. 10.1016/j.bbamem.2007.05.025 17618864

[B211] Mukherjee S. Raghuraman H. Chattopadhyay A. (2007b). Membrane localization and dynamics of Nile Red: effect of cholesterol. Biochim. Biophys. Acta 1768, 59–66. 10.1016/j.bbamem.2006.07.010 16934217

[B212] Mukherjee S. Raghuraman H. Dasgupta S. Chattopadhyay A. (2004). Organization and dynamics of *N*-(7-nitrobenz-2-oxa-1,3-diazol-4-yl)-labeled lipids: a fluorescence approach. Chem. Phys. Lipids 127, 91–101. 10.1016/j.chemphyslip.2003.09.004 14706743

[B213] Murata Y. Iwasaki H. Sasaki M. Inaba K. Ikamura Y. (2005). Phosphoinositide phosphatase activity coupled to an intrinsic voltage sensor. Nature 435, 1239–1243. 10.1038/nature03650 15902207

[B214] Musse A. A. Wang J. Deleon G. P. Prentice G. A. London E. Merrill A. R. (2006). Scanning the membrane-bound conformation of helix 1 in the colicin E1 channel domain by site-directed fluorescence labeling. J. Biol. Chem. 281, 885–895. 10.1074/jbc.M511140200 16299381

[B215] Nagahama M. Mukai M. Morimitsu S. Ochi S. Sakurai J. (2002). Role of the c-domain in the biological activities of *Clostridium perfringens* alphs-toxin. Microbiol. Immunol. 46, 647–655. 10.1111/j.1348-0421.2002.tb02748.x 12477243

[B216] Nguyen A. H. Nguyen V. T. Kamio Y. Higuchi H. (2006). Single-molecule visualization of environment-sensitive fluorophores inserted into cell membranes by Staphylococcal γ-hemolysin. Biochemistry 45, 2570–2576. 10.1021/bi0514156 16489750

[B217] Noskov S. Y. Roux B. (2007). Importance of hydration and dynamics on the selectivity of the KcsA and NaK channels. J. Gen. Physiol. 129, 433–445. 10.1085/jgp.200609633 17227917 PMC2154357

[B218] Ohsugi Y. Saito K. Tamura M. Kinjo M. (2006). Lateral mobility of membrane-binding proteins in living cells measured by total internal reflection fluorescence correlation spectroscopy. Biophys. J. 91, 3456–3464. 10.1529/biophysj.105.074625 16891361 PMC1614500

[B219] Olofsson A. Herbert H. Thelestam M. (1993). The projection structure of perfringolysin-O (*Clostridium perfringens* theta-toxin). FEBS Lett. 319, 125–127. 10.1016/0014-5793(93)80050-5 8454043

[B220] Opella S. J. (1997). NMR and membrane proteins. *Nat*. Struct. Biol. 4, 845–848. 9377156

[B221] Ostmeyer J. Chakrapani S. Pan A. C. Perozo E. Roux B. (2013). Recovery from slow inactivation in K^+^ channels is controlled by water molecules. Nature 501, 121–124. 10.1038/nature12395 23892782 PMC3799803

[B222] Palmer L. R. Merrill A. R. (1994). Mapping the membrane topology of the closed state of the colicin E1 channel. J. Biol. Chem. 269, 4187–4193. 7508440

[B223] Parker M. W. Feil S. C. (2005). Pore-forming toxins: from structure to function. Prog. Biophys. Mol. Biol. 88, 91–142. 10.1016/j.pbiomolbio.2004.01.009 15561302

[B224] Pathak M. M. Yarov-Yarovoy V. Agarwal G. Roux B. Barth P. Kohout S. Isacoff E. Y.. (2007). Closing in on the resting state of the Shaker K^+^ channel. Neuron 56, 124–140. 10.1016/j.neuron.2007.09.023 17920020

[B225] Patowary S. Alvarez-Curto E. Xu T. R. Holz J. D. Oliver J. A. Milligan G. Raicu V. (2013). The muscarinic M3 acetylcholine receptor exists as two differently sized complexes at the plasma membrane. Biochem. J. 452, 303–312. 10.1042/BJ20121902 23521066

[B226] Pattnaik B. R. Ghosh S. Rajeswari M. R. (1997). Selective excitation of tryptophans in OmpF: a fluorescence emission study. Biochem. Mol. Biol. Int. 42, 173–181. 10.1080/15216549700202561 9192098

[B227] Perier A. Gourier C. Pichard S. Husson J. Lajeunesse E. Babon A. Gillet D.. (2007). Creation of intercellular bonds by anchoring protein ligands to membranes using the diphtheria toxin T domain. FEBS Lett. 581, 5480–5544. 10.1016/j.febslet.2007.10.055 17991440

[B228] Perozo E. Cuello L. G. Cortes D. M. Liu Y. S. Sompornpisut P. (2002). EPR approaches to ion channel structure and function. Novartis Found. Symp. 245, 146–158. 10.1002/0470868759.ch10 12027005

[B229] Pick H. Preuss A. K. Mayer M. Wohland T. Hovius R. Vogel H. (2003). Monitoring expression and clustering of the ionotropic 5HT_3_ receptor in plasma membranes of live biological cells. Biochemistry 42, 877–884. 10.1021/bi026576d 12549905

[B230] Piston D. W. Kremers G.-J. (2007). Fluorescent protein FRET: the good, the bad and the ugly. Trends Biochem. Sci. 32, 407–414. 10.1016/j.tibs.2007.08.003 17764955

[B231] Posokhov Y. O. Rodnin M. V. Das S. K. Pucci B. Ladokhin A. S. (2008a). FCS study of the thermodynamics of membrane protein insertion into the lipid bilayer chaperoned by fluorinated surfactants. Biophys. J. 95:L54–56. 10.1529/biophysj.108.141002 18708456 PMC2553139

[B232] Posokhov Y. O. Rodnin M. V. Lu L. Ladokhin A. S. (2008b). Membrane insertion pathway of annexin B12: thermodynamic and kinetic characterization by fluorescence correlation spectroscopy and fluorescence quenching. Biochemistry 47, 5078–5087. 10.1021/bi702223c 18407663

[B233] Powl A. M. Wright J. N. East J. M. Lee A. G. (2005). Identification of the hydrophobic thickness of a membrane protein using fluorescence spectroscopy: studies with the mechanosensitive channel MscL. Biochemistry 44, 5713–5721. 10.1021/bi047338g 15823029

[B234] Prendergast F. G. (1991). Time-resolved fluorescence techniques: methods and applications in biology. Curr. Opin. Struct. Biol. 1, 1054–1059. 10.1016/0959-440X(91)90105-3

[B235] Puljung M. C. Zagotta W. N. (2011). Labeling of specific cysteines in proteins using reversible metal protection. Biophys. J. 100, 2513–2521. 21575586 10.1016/j.bpj.2011.03.063 PMC3093568

[B236] Raghuraman H. Chattopadhyay A. (2003). Organization and dynamics of melittin in environments of graded hydration: a fluorescence approach. Langmuir 19, 10332–10341. 10.1021/la035126z

[B237] Raghuraman H. Chattopadhyay A. (2004a). Effect of micellar charge on the conformation and dynamics of melittin. Eur. Biophys. J. 33, 611–622. 10.1007/s00249-004-0402-7 15071759

[B238] Raghuraman H. Chattopadhyay A. (2004b). Influence of lipid chain unsaturation on membrane-bound melittin: a fluorescence approach. Biochim. Biophys. Acta 1665, 29–39. 10.1016/j.bbamem.2004.06.008 15471568

[B239] Raghuraman H. Chattopadhyay A. (2004c). Interaction of melittin with membrane cholesterol: a fluorescence approach. Biophys. J. 87, 2419–2432. 10.1529/biophysj.104.043596 15454440 PMC1304663

[B240] Raghuraman H. Chattopadhyay A. (2005). Cholesterol inhibits the lytic activity of melittin in erythrocytes. Chem. Phys. Lipids 134, 183–189. 10.1016/j.chemphyslip.2004.12.011 15784236

[B241] Raghuraman H. Chattopadhyay A. (2006). Effect of ionic strength on folding and aggregation of the hemolytic peptide melittin in solution. Biopolymers 83, 111–121. 10.1002/bip.20536 16680713

[B242] Raghuraman H. Chattopadhyay A. (2007a). Melittin: a membrane-active peptide with diverse functions. Biosci. Rep. 27, 189–223. 10.1007/s10540-006-9030-z 17139559

[B243] Raghuraman H. Chattopadhyay A. (2007b). Orientation and dynamics of melittin in membranes of varying composition utilizing NBD fluorescence. Biophys. J. 92, 1271–1283. 10.1529/biophysj.106.088690 17114219 PMC1783871

[B244] Raghuraman H. Cordero-Morales J. F. Jogini V. Pan A. C. Kollewe A. Roux B. Perozo E. (2012). Mechanism of Cd^2+^ coordination during slow inactivation in potassium channels. Structure 20, 1332–1342. 10.1016/j.str.2012.03.027 22771214 PMC3846092

[B245] Raghuraman H. Ganguly S. Chattopadhyay A. (2006). Effect of ionic strength on the organization and dynamics of membrane-bound melittin. Biophys. Chem. 124, 115–124. 10.1016/j.bpc.2006.06.011 16831504

[B246] Raghuraman H. Islam S. M. Mukherjee S. Roux B. Perozo E. (2014). Dynamics transitions at the outer vestibule of the KcsA potassium channel during gating. Proc. Natl. Acad. Sci. U.S.A. 111, 1831–1836. 10.1073/pnas.1314875111 24429344 PMC3918809

[B247] Raghuraman H. Kelkar D. A. Chattopadhyay A. (2003). Novel insights into membrane protein structure and dynamics utilizing the wavelength-selective fluorescence approach. Proc. Indian Natl. Sci. Acad. A 69, 25–35.

[B248] Raghuraman H. Kelkar D. A. Chattopadhyay A. (2005). Novel insights into protein structure and dynamics utilizing the red edge excitation shift approach, in Reviews in Fluorescence, eds Geddes C. D. Lakowicz J. R. (New York, NY: Springer, 199–214.

[B249] Raghuraman H. Pradhan S. K. Chattopadhyay A. (2004). Effect of urea on the organization and dynamics of Triton X-100 micelles: a fluorescence approach. J. Phys. Chem. B 108, 2489–2496. 10.1021/jp0365007

[B250] Raghuraman H. Shrivastava S. Chattopadhyay A. (2007). Monitoring the looping up of acyl chain labeled NBD lipids in membranes as a function of membrane phase state. Biochim. Biophys. Acta 1768, 1258–1267. 10.1016/j.bbamem.2007.02.001 17362875

[B251] Raicu V. Singh D. R. (2013). FRET spectroscopy: a new tool for the determination of protein quaternary structure in living cells. Biophys. J. 105, 1937–1945. 10.1016/j.bpj.2013.09.015 24209838 PMC3824708

[B252] Ramachandran R. Tweten R. K. Johnson A. E. (2004). Membrane-dependent conformational changes initiate cholesterol-dependent cytolysin oligomerization and intersubunit beta-strand alignment. Nat. Struct. Mol. Biol. 11, 697–705. 10.1038/nsmb793 15235590

[B253] Ramalingam T. S. Das P. K. Podder S. K. (1994). Ricin-membrane interaction: membrane penetration depth by fluorescence quenching and resonance energy transfer. Biochemistry 33, 12247–12254. 10.1021/bi00206a030 7918445

[B254] Rawat S. S. Kelkar D. A. Chattopadhyay A. (2004). Monitoring gramicidin conformations in membranes: a fluorescence approach. Biophys. J. 87, 831–843. 10.1529/biophysj.104.041715 15298892 PMC1304493

[B255] Renart M. L. Guidici A. M. Poveda J. A. Fedorov A. Berberan-Santos M. N. Prieto M.. (2019). Conformational plasticity in the KcsA potassium channel pore helix revealed by homo-FRET studies. Sci. Rep. 9:6215. 10.1038/s41598-019-42405-5 30996281 PMC6470172

[B256] Rhoades E. Ramlall T. F. Webb W. W. Eliezer D. (2006). Quantification of alpha-synuclein binding to lipid vesicles using fluorescence correlation spectroscopy. Biophys. J. 90, 4692–4700. 10.1529/biophysj.105.079251 16581836 PMC1471845

[B257] Richardson J. Blunck R. Ge P. Selvin P. R. Bezanilla F. Papazian D. M. Correa A. M. (2006). Distance measurements reveal a common topology of prokaryotic voltage-gated ion channels in the lipid bilayer. Proc. Natl. Acad. Sci. U.S.A. 103, 15865–15870. 10.1073/pnas.0607532103 17043236 PMC1635094

[B258] Ries J. Schwille P. (2012). Fluorescence correlation spectroscopy. Bioessays 34, 361–368. 10.1002/bies.201100111 22415816

[B259] Rodriguez E. A. Campbell R. E. Lin J. Y. Lin M. Z. Miyawaki A. Palmer A. E.. (2017). The growing and glowing toolbox of fluorescent and photoactive proteins. Trends Biochem. Sci. 42, 111–129. 10.1016/j.tibs.2016.09.010 27814948 PMC5272834

[B260] Roy R. Hohng S. Ha T. (2008). A practical guide to single-molecule FRET. Nat. Methods 5, 507–516. 10.1038/nmeth.1208 18511918 PMC3769523

[B261] Rusu L. Gambhir A. McLaughlin S. Rädler J. (2004). Fluorescence correlation spectroscopy studies of peptide and protein binding to phospholipid vesicles. Biophys. J. 87, 1044–1053. 10.1529/biophysj.104.039958 15298909 PMC1304445

[B262] Sadler E. E. Kapanidis A. N. Tucker S. J. (2016). Solution-based single-molecule FRET studies of K^+^ channel gating in a lipid bilayer. Biophys. J. 110, 2663–2670. 10.1016/j.bpj.2016.05.020 27332124 PMC4919593

[B263] Sakon J. J. Weninger K. R. (2010). Detecting the conformation of individual proteins in live cells. Nat.Methods 7, 203–205. 10.1038/nmeth.1421 20118931 PMC2844853

[B264] Sandtner W. Bezanilla F. Correa A. M. (2007). *In vivo* measurement of intramolecular distances using genetically encoded reporters. Biophys. J. 93:L45–L47. 10.1529/biophysj.107.119073 17766346 PMC2025654

[B265] Sapsford K. E. Berti L. Medintz I. L. (2006). Materials for fluorescence resonance energy transfer analysis: beyond traditional donor-acceptor combinations. Angew. Chem. Int. Ed. 45, 4562–4588. 10.1002/anie.200503873 16819760

[B266] Sarkar P. Chattopadhyay A. (2019). Exploring membrane organization at varying spatiotemporal resolutions utilizing fluorescence-based approaches: implications in membrane biology. Phys. Chem. Chem. Phys. 21, 11554–11563. 10.1039/C9CP02087J 31134261

[B267] Sasmal D. K. Lu H. P. (2014). Single-molecule patch-clamp FRET microscopy studies of NMDA receptor ion channel dynamics in living cells: revealing the multiple conformational states associated with a channel at its electrical off state. J. Am. Chem. Soc. 136, 12998–13005. 10.1021/ja506231j 25148304 PMC4183623

[B268] Sasmal D. K. Pulido L. E. Kasal S. Huang J. (2016a). Single-molecule fluorescence resonance energy transfer in molecular biology. Nanoscale 8, 19928–19944. 10.1039/C6NR06794H 27883140 PMC5145784

[B269] Sasmal D. K. Yadav R. Lu H. P. (2016b). Single-molecule patch-clamp FRET anisotropy microscopy studies of NMDA receptor ion channel activation and deactivation under agonist ligand binding in living cells. J. Am. Chem. Soc. 138, 8789–8801. 10.1021/jacs.6b03496 27270213

[B270] Schick S. Chen L. Li E. Lin J. Koper I. Hristova K. (2010). Assembly of the M2 tetramer is strongly modulated by lipid chain length. Biophys. J. 99, 1810–1817. 10.1016/j.bpj.2010.07.026 20858425 PMC2941016

[B271] Schiffer M. Chang C. H. Stevens F. J. (1992). The functions of tryptophan residues in membrane proteins. Protein Eng. 5, 213–214. 10.1093/protein/5.3.213 1409540

[B272] Schirmer T. Keller T. A. Wang Y. F. Rosenbusch J. P. (1995). Structural basis for sugar translocation through maltoporin channels at 3.1-angstrom resolution. Science 267, 512–514. 10.1126/science.7824948 7824948

[B273] Schroder G. F. Alexiev U. Grubmuller H. (2005). Simulation of fluorescence anisotropy experiments: probing protein dynamics. Biophys. J. 89, 3757–3770. 10.1529/biophysj.105.069500 16169987 PMC1366944

[B274] Schwille P. Haupts U. Maiti S. Webb W. W. (1999a). Molecular dynamics in living cells observed by fluorescence correlation spectroscopy with one- and two-photon excitation. Biophys. J. 77, 2251–2265. 10.1016/S0006-3495(99)77065-7 10512844 PMC1300505

[B275] Schwille P. Korlach J. Webb W. W. (1999b). Fluorescence correlation spectroscopy with single-molecule sensitivity on cell and model membranes. Cytometry 36:176–182. 10404965 10.1002/(sici)1097-0320(19990701)36:3<176::aid-cyto5>3.0.co;2-f

[B276] Selvin P. R. (1995). Fluorescence resonance energy transfer. Meth. Enzymol. 246, 300-334. 10.1016/0076-6879(95)46015-2 7752929

[B277] Semenova N. P. Abarca-Heidemann K. Loranc E. Rothberg B. S. (2009). Bimane fluorescence scanning suggests secondary structure near the S3-S4 linker of BK channels. J. Biol. Chem. 284, 10684–10693. 10.1074/jbc.M808891200 19244238 PMC2667755

[B278] Shai Y. (2001). Molecular recognition within the membrane milieu: implications for the structure and function proteins. J Membr. Biol. 182, 91–104. 10.1007/s00232-001-0034-a 11447501

[B279] Shatursky O. Heuck A. P. Shepard L. A. Rossjohn J. Parker M. W. Johnson A. E.. (1999). The mechanism of membrane insertion for a cholesterol-dependent cytolysin: a novel paradigm for pore-forming toxins. Cell 99, 293–299. 10.1016/S0092-8674(00)81660-8 10555145

[B280] Shepard L. A. Heuck A. P. Hamman B. D. Rossjohn J. Parker M. W. Ryan K. R.. (1998). Identification of a membrane-spanning domain of the thiol-activated pore-forming toxin *Clostridium perfringens* perfringolysin O: an α-helical to β-sheet transition identified by fluorescence spectroscopy. Biochemistry 37, 14563–14574. 10.1021/bi981452f 9772185

[B281] Singh D. R. Mohammad M. M. Patowary S. Stoneman M. R. Oliver J. A. Movileanu L.. (2013). Determination of the quaternary structure of a bacterial ATP-binding cassette (ABC) transporter in living cells. Integr. Biol. 5, 312–323. 10.1039/c2ib20218b 23223798 PMC3558595

[B282] Smith D. A. McKenzie G. Jones A. C. Smith T. A. (2017). Analysis of time-correlated single photon counting data: a comparative evaluation of deterministic and probabilistic approaches. Methods Appl. Fluoresc. 5, 042001. 10.1088/2050-6120/aa8055 29063861

[B283] Stryer L. (1978). Fluorescence energy transfer as a spectroscopic ruler. Annu. Rev. Biochem. 47, 829–846. 10.1146/annurev.bi.47.070178.004131 354506

[B284] Sun M. Song P. S. (1977). Solvent effects on the fluorescence states of indole derivatives- dipole moments. Photochem. Photobiol. 25, 3–9. 10.1111/j.1751-1097.1977.tb07416.x

[B285] Sun Y. Rombola C. Jyothikumar V. Periasamy A. (2013). Förster resonance energy transfer microscopy and spectroscopy for localizing protein-protein interactions in living cells. Cytometry A. 83, 780–793. 10.1002/cyto.a.22321 23813736 PMC3924896

[B286] Tan Y.-W. Hanson J. A. Chu J.-W. Yang H. (2014). Confocal single-molecule FRET for protein conformational dynamics, in Protein Dynamics: Methods and Protocols (Methods in Molecular Biology), Vol. 1084, ed Livesay D. R. (New York, NY: Springer Science+Business Media, 51–62. 10.1007/978-1-62703-658-0_3 24061915

[B287] Taraska J. W. (2012). Mapping membrane protein structure with fluorescence. Curr. Opin. Struct. Biol. 22, 507–513. 10.1016/j.sbi.2012.02.004 22445227 PMC3498957

[B288] Taraska J. W. Puljung M. C. Olivier N. B. Flynn G. E. Zagotta W. N. (2009b). Mapping the structure and conformational movements of proteins with transition metal ion FRET. Nat. Methods 6, 532–537. 10.1038/nmeth.1341 19525958 PMC2738593

[B289] Taraska J. W. Puljung M. C. Zagotta W. N. (2009a). Short-distance probes for protein backbone structure based on energy transfer between bimane and transition metal ions. Proc. Natl. Acad. Sci. U.S.A. 10, 16227–16232. 10.1073/pnas.0905207106 PMC2741476 19805285

[B290] Terry D. S. Kolster R. A. Quick M. LeVine M. V. Khelashvili G. Zhou Z. (2018). A partially-open inward-facing intermediate conformation of LeuT is associated with Na^+^ release and substrate transport. Nat. Commun. 15:230 10.1038/s41467-017-02202-y PMC5768729 29335402

[B291] Terstappen G. C. Reggiani A. (2001). *In silico* research in drug discovery. Trends Pharmacol. Sci. 22, 23–26. 10.1016/S0165-6147(00)01584-4 11165668

[B292] Thomas D. D. (1978). Large-scale rotational motions of proteins detected by electron paramagnetic resonance and fluorescence. Biophys. J. 24, 439–462. 10.1016/S0006-3495(78)85394-6 215240 PMC1473419

[B293] Thompson N. L. Axelrod D. (1983). Immunoglobulin surface-binding kinetics studied by total internal reflection with fluorescence correlation spectroscopy. Biophys. J. 43, 103–114. 10.1016/S0006-3495(83)84328-8 6882857 PMC1329273

[B294] Tilley S. J. Orlova E. V. Gilbert R. J. C. Andrew P. W. Saibil H. R. (2005). Structural basis of pore formation by the bacterial toxin pneumolysin. Cell 121, 247–256. 10.1016/j.cell.2005.02.033 15851031

[B295] Tombola F. Ulbrich M. H. Kohout S. C. Isacoff E. Y. (2010). The opening of the two pores of the Hv1 voltage-gated proton channel is tuned by cooperativity? Nat. Struct. Mol. Biol. 17, 44–50. 10.1038/nsmb.1738 20023640 PMC2925041

[B296] Tomita A. Zhang M. Jin F. Zhuang W. Takeda H. Maruyama T.. (2017). ATP-dependent modulation of MgtE in Mg^2+^ homeostasis. Nat. Commun. 8:148. 10.1038/s41467-017-00082-w 28747715 PMC5529423

[B297] Torbeev V. Yu. Raghuraman H. Hamelberg D. Tonelli M. Westler W. M. Perozo E.. (2011). Protein conformational dynamics in the mechanism of HIV-1 protease catalysis. Proc. Natl. Sci. Acad. U.S.A. 108, 20982–20987. 10.1073/pnas.1111202108 22158985 PMC3248522

[B298] Torbeev V. Yu. Raghuraman H. Mandal K. Senapati S. Perozo E. Kent S. B. H. (2009). Dynamics of “flap” structures in three HIV-1 protease/inhibitor complexes probed by total chemical synthesis and pulsed-EPR spectroscopy. J. Am. Chem. Soc. 131, 884–885. 10.1021/ja806526z 19117390

[B299] Tory M. C. Merrill A. R. (1999). Adventures in membrane protein topology: a study of the membrane-bound state of colicin E1. J. Biol. Chem. 274, 24539–24549. 10.1074/jbc.274.35.24539 10455117

[B300] Tory M. C. Merrill A. R. (2002). Determination of membrane protein topology by red-edge excitation shift analysis: application to the membrane-bound colicin E1 channel peptide. Biochim. Biophys. Acta 1564, 435–448. 10.1016/S0005-2736(02)00493-5 12175927

[B301] Tsukamoto H. Farrens D. L. (2013). A constitutively activating mutation alters the dynamics and energetics of a key conformational change in a ligand-free G protein-coupled receptor. J. Biol. Chem. 288, 28207–28216. 10.1074/jbc.M113.472464 23940032 PMC3784730

[B302] Tsukamoto H. Farrens D. L. Koyanagi M. Terakita A. (2009). The magnitude of the light-induced conformational change in different rhodopsins correlates with their ability to activate G proteins. J. Biol. Chem. 284, 20676–20683. 10.1074/jbc.M109.016212 19497849 PMC2742832

[B303] Turconi S. Bingham R. P. Haupts U. Pope A. J (2001). Developments in fluorescence lifetime-based analysis for ultra-HTS. Drug Discov. Today 6, S27–S39. 10.1016/S1359-6446(01)00159-3

[B304] Ulmschneider M. B. Sansom M. S. P. (2001). Amino acid distributions in integral membrane protein structures. Biochim. Biophys. Acta 1512, 1–14. 10.1016/S0005-2736(01)00299-1 11334619

[B305] Vafabakhsh R. Levitz J. Isacoff E. Y. (2015). Conformational dynamics of a class C G-protein-coupled receptor. Nature 524, 497–501. 10.1038/nature14679 26258295 PMC4597782

[B306] Van Der Meer B. W. Coker G. I. I. I. Chen S.-Y. S. (1994). Resonance Energy Transfer: Theory and Data. New York, NY: VCH.

[B307] Vargas-Uribe M. Rodnin M. V. Ladokhin A. S. (2013). Comparison of membrane insertion pathways of the apoptotic regulator Bcl-xL and the diphtheria toxin translocation domain. Biochemistry 52, 7901–7909. 10.1021/bi400926k 24134052 PMC3882133

[B308] Varma R. Mayor S. (1998). GPI-anchored proteins are organized in submicron domains on the cell surface. Nature 394, 798–801. 10.1038/29563 9723621

[B309] Veatch W. Stryer L. (1977). The dimeric nature of the gramicidin A transmembrane channel: conductance and fluorescence energy transfer studies of hybrid channels. J. Mol. Biol. 113, 89–102. 10.1016/0022-2836(77)90042-0 69713

[B310] Verhalen B. Ernst S. Börsch M. Wilkens S. (2012). Dynamic ligand-induced conformational rearrangements in P-glycoprotein as probed by fluorescence resonance energy transfer spectroscopy. J. Biol. Chem. 287, 1112–1127. 10.1074/jbc.M111.301192 22086917 PMC3256922

[B311] Vicidomini G. Ta H. Honigmann A. Mueller V. Clausen M. P. Waithe D.. (2015). STED-FLCS: an advanced tool to reveal spatiotemporal heterogeneity of molecular membrane dynamics. Nano Lett. 15, 5912–5918. 10.1021/acs.nanolett.5b02001 26235350 PMC4819494

[B312] Villalba-Galea C. A. Miceli F. Taglialatela M. Bezanilla F. (2009). Coupling between the voltage-sensing and phosphatase domains of Ci-VSP. J. Gen. Physiol. 134, 5–14. 10.1085/jgp.200910215 19564425 PMC2712979

[B313] Vivian J. T. Callis P. R. (2001). Mechanisms of tryptophan fluorescence shifts in proteins. Biophys. J. 80, 2093–2109. 10.1016/S0006-3495(01)76183-8 11325713 PMC1301402

[B314] Volz P. Krause N. Balke J. Schneider C. Walter M. Schneider F.. (2016). Light and pH-induced changes in structure and accessibility of transmembrane helix B and its immediate environment in Channelrhodopsin-2. J. Biol. Chem. 291, 17382–17393. 10.1074/jbc.M115.711200 27268055 PMC5016135

[B315] Wallace B. A. Janes R. W. (1999). Tryptophans in membrane proteins, in Tryptophan, Serotonin, and Melatonin: Basic Aspects and Applications (Advances in Experimental Medicine and Biology, Vol.467), eds Huether G. Kochen W. Simat T. J. Steinhart H. (New York, NY: Springer Science+Business Media, 789–799. 10.1007/978-1-4615-4709-9_101 10721132

[B316] Wallin E. Heijne G. V. (1998). Genome-wide analysis of integral membrane proteins from eubacterial, archaean, and eukaryotic organisms. Protein Sci. 7, 1029–1038. 10.1002/pro.5560070420 9568909 PMC2143985

[B317] Wang S. Brettmann J. B. Nichols C. G. (2018). Studying structural dynamics of potassium channels by single-molecule FRET, in Potassium Channels: Methods and Protocols, Methods in Molecular Biology, eds Shyng S.-L. Valiyaveetil F. I. Whorton M. (New York, NY: Springer Science+Business Media, LLC, 163–180. 10.1007/978-1-4939-7362-0_13 PMC6317966 29058191

[B318] Wang S. Lee S.-J. Maksaev G. Fang X. Zuo C. Nichols C. G. (2019). Potassium channel selectivity filter dynamics revealed by single-molecule FRET. Nat. Chem. Biol. 15, 377–383. 10.1038/s41589-019-0240-7 30833778 PMC6430689

[B319] Wang S. Vafabakhsh R. Borschel W. F. Ha T. Nichols C. G. (2016). Structural dynamics of potassium-channel gating revealed by single-molecule FRET. Nat. Struct. Mol. Biol. 23, 31–36. 10.1038/nsmb.3138 26641713 PMC4833211

[B320] Wang Y. Liu Y. Deberg H. A. Nomura T. Hoffman M. T. Rohde P. R.. (2014). Single molecule FRET reveals pore size and opening mechanism of a mechano-sensitive ion channel. Elife 3:e01834. 10.7554/eLife.01834 24550255 PMC3925968

[B321] Weber G. (1960a). Fluorescence-polarization spectrum and electronic-energy transfer in tyrosine, tryptophan and related compounds. Biochem. J. 75, 335–345. 10.1042/bj0750335 13843297 PMC1204430

[B322] Weber G. (1960b). Fluorescence-polarization spectrum and electronic-energy transfer in proteins. Biochem. J. 75, 345–352. 10.1042/bj0750345 13843296 PMC1204431

[B323] Weber G. Shinitsky M. (1970). Failure of energy transfer between identical aromatic molecules on excitation at the long wave edge of the absorption spectrum. Proc. Natl. Sci. Acad. U.S.A. 65, 823–830. 10.1073/pnas.65.4.823 16591825 PMC282989

[B324] Wei Z. White D. Wang J. Musse A. A. Merrill A. R. (2007). Tilted, extended, and lying in wait: the membrane-bound topology of residues Lys-381-Ser-405 of the colicin E1 channel domain. Biochemistry 46, 6074–6085. 10.1021/bi700317k 17455912

[B325] Weiss S. (2000). Measuring conformational dynamics of biomolecules by single molecule fluorescence spectroscopy. Nat. Struct. Biol. 7, 724–729. 10.1038/78941 10966638

[B326] White A. E. Raghuraman H. Perozo E. Glotzer M. (2013). Binding of the CYK-4 subunit of the centralspindlin complex induces a large scale conformational change in the kinesin subunit. J. Biol. Chem. 288, 19785–19795. 10.1074/jbc.M113.463695 23720745 PMC3707682

[B327] White S. H. Wimley W. C. (1994). Peptides in lipid bilayers: structural and thermodynamic basis for partitioning and folding. Curr. Opin. Struct. Biol. 4, 79–86. 10.1016/S0959-440X(94)90063-9

[B328] Wu P. Brand L. (1994). Resonance energy transfer: methods and applications. Anal. Biochem. 218, 1–13. 10.1006/abio.1994.1134 8053542

[B329] Wüstner D. Mukherjee S. Maxfield F. R. Müller P. Hermann A. (2001). Vesicular and nonvesicular transport of phosphatidylcholine in polarized HepG2 cells. Traffic 2, 277–296. 10.1034/j.1600-0854.2001.9o135.x 11285138

[B330] Yao X. Parnot C. Deupi X. Ratnala V. R. Swaminath G. Farrens D. Kobilka B. (2006). Coupling ligand structure to specific conformational switches in the beta2-adrenoceptor. Nat. Chem. Biol. 2, 417–422. 10.1038/nchembio801 16799554

[B331] Yau W.-M. Wimley W. C. Gawrisch K. White S. H. (1998). The preference of tryptophan for membrane interfaces. Biochemistry 37, 14713–14718. 10.1021/bi980809c 9778346

[B332] Yildirim M. A. Goh K.-I. Cusick M. E. Barabsai A.-L. Vidal M. (2007). Drug-target network. Nat. Biotechnol. 25, 1119–1126. 10.1038/nbt1338 17921997

[B333] Yu X. Wu X. Bermejo G. A. Brooks B. R. Taraska J. W. (2013). Accurate high-throughput structure mapping and prediction with transition metal ion FRET. Structure 21, 9–19. 10.1016/j.str.2012.11.013 23273426 PMC3700372

[B334] Zhang Z. Yomo D. Gradinaru C. (2017). Choosing the right fluorophore for single-molecule fluorescence studies in a lipid environment. Biochim. Biophys. Acta 1859, 1242–1253. 10.1016/j.bbamem.2017.04.001 28392350

[B335] Zhao Y. Terry D. Shi L. Weinstein H. Blanchard S. C. Javitch J. A. (2010). Single-molecule dynamics of gating in a neurotransmitter transporter homologue. Nature 465, 188–193. 10.1038/nature09057 20463731 PMC2940119

[B336] Zheng J. Zagotta W. N. (2000). Gating rearrangements in cyclic nucleotide-gated channels revealed by patch-clamp fluorometry. Neuron 28, 369–374. 10.1016/S0896-6273(00)00117-3 11144348

[B337] Zheng J. Zagotta W. N. (2003). Patch-clamp fluorometry recording of conformational rearrangements of ion channels. Sci STKE 176:PL7 10.1126/stke.2003.176.pl7 12671191

[B338] Zhou H.-X. Cross T. A. (2013). Influences of membrane mimetic environments on membrane protein structures. Annu. Rev. Biophys. 42, 16.1-16.32. 10.1146/annurev-biophys-083012-130326 23451886 PMC3731949

[B339] Zhou Y. Morais-Cabral J. H. Kaufman A. MacKinnon R. (2001). Chemistry of ion coordination and hydration revealed by a K^+^ channel-Fab complex at 2.0 Å resolution. Nature 414, 43–48. 10.1038/35102009 11689936

[B340] Zoldak G. Jancura D. Sedlak E. (2017). The fluorescence intensities ratio is not a reliable parameter for evaluation of protein unfolding transitions. Protein Sci. 26, 1236–1239. 10.1002/pro.3170 28370732 PMC5441425

